# Potentiality of Actinomycetia Prevalent in Selected Forest Ecosystems in Assam, India to Combat Multi-Drug-Resistant Microbial Pathogens

**DOI:** 10.3390/metabo13080911

**Published:** 2023-08-03

**Authors:** Rajkumari Mazumdar, Kangkon Saikia, Debajit Thakur

**Affiliations:** 1 Microbial Biotechnology Laboratory, Life Sciences Division, Institute of Advanced Study in Science and Technology (IASST), Guwahati 781035, India; rajkumarimazumdar@gmail.com; 2 Department of Molecular Biology & Biotechnology, Cotton University, Guwahati 781001, India; 3 Bioinformatics Infrastructure Facility, Institute of Advanced Study in Science and Technology, Guwahati 781035, India; kangkonsaikia@gmail.com

**Keywords:** actinomycetia, antibiotics, MDR, UTI, secondary metabolites, natural product, genome mining, biosynthetic gene clusters, WGS

## Abstract

Actinomycetia are known for their ability to produce a wide range of bioactive secondary metabolites having significant therapeutic importance. This study aimed to explore the potential of actinomycetia as a source of bioactive compounds with antimicrobial properties against multi-drug-resistant (MDR) clinical pathogens. A total of 65 actinomycetia were isolated from two unexplored forest ecosystems, namely the Pobitora Wildlife Sanctuary (PWS) and the Deepor Beel Wildlife Sanctuary (DBWS), located in the Indo-Burma mega-biodiversity hotspots of northeast India, out of which 19 isolates exhibited significant antimicrobial activity. 16S rRNA gene sequencing was used for the identification and phylogenetic analysis of the 19 potent actinomycetia isolates. The results reveal that the most dominant genus among the isolates was *Streptomyces* (84.21%), followed by rare actinomycetia genera such as *Nocardia*, *Actinomadura*, and *Nonomuraea*. Furthermore, seventeen of the isolates tested positive for at least one antibiotic biosynthetic gene, specifically type II polyketide synthase (PKS-II) and nonribosomal peptide synthetases (NRPSs). These genes are associated with the production of bioactive compounds with antimicrobial properties. Among the isolated strains, three actinomycetia strains, namely *Streptomyces* sp. PBR1, *Streptomyces* sp. PBR36, and *Streptomyces* sp. DBR11, demonstrated the most potent antimicrobial activity against seven test pathogens. This was determined through in vitro antimicrobial bioassays and the minimum inhibitory concentration (MIC) values of ethyl acetate extracts. Gas chromatography–mass spectrometry (GS-MS) and whole-genome sequencing (WGS) of the three strains revealed a diverse group of bioactive compounds and secondary metabolite biosynthetic gene clusters (smBGCs), respectively, indicating their high therapeutic potential. These findings highlight the potential of these microorganisms to serve as a valuable resource for the discovery and development of novel antibiotics and other therapeutics with high therapeutic potential.

## 1. Introduction

Antimicrobial drugs have revolutionized modern medicine by facilitating medical procedures, such as advanced surgical techniques, organ transplantation, and cancer treatment [1]. Rapid prevalence of antibiotic resistance has emerged as a major health concern, posing substantial risk to therapeutic interventions and resulting in approximately 700,000 deaths worldwide each year. In particular, the majority of these deaths were reported mainly in low- and low–middle-income countries (LMICs) [2]. Therefore, it is crucial to comprehend the primary pathogen–drug combinations that contribute to the current level of antimicrobial resistance (AMR) and their prevalence. In 2014, researchers estimated the occurrence of *Escherichia coli* and *Klebsiella pneumoniae* resistance to third-generation cephalosporins and carbapenems in 193 countries [3,4]. Six bacterial pathogens, namely *E. coli*, *Staphylococcus aureus*, *Klebsiella pneumoniae*, *Streptococcus pneumoniae*, *Acinetobacter baumannii*, and *Pseudomonas aeruginosa*, were responsible for over 250,000 deaths in 2019 and have been designated as priority pathogens by the World Health Organization (WHO) [3]. Globally, there have been high rates of resistance to antibiotics commonly used for treating bacterial infections, such as urinary tract infections (UTIs), sepsis, and sexually transmitted infections, observed in clinical settings [5]. Consequently, the treatment of antibiotic resistance has become increasingly challenging, necessitating the development of new antimicrobial drugs [6]. Thus, alternative strategies to mitigate antibiotic resistance have become an urgent requirement. In this context, the vast array of antimicrobial agents produced by actinomycetia plays a pivotal role in combating the antibiotic resistance crisis [7].

Actinomycetia, formerly known as actinobacteria, are filamentous bacteria that are Gram-positive and have a high Guanine and Cytosine (G + C) content in their genome. These bacteria are well known for their ability to produce bioactive secondary metabolites [8]. Among the actinomycetia, the *Streptomyces* species is particularly noteworthy, as they alone produce approximately 7600 bioactive compounds [9]. The genome of *Streptomyces* sp. typically contains 20 to 40 different types of biosynthetic gene clusters (BGCs). These gene clusters are responsible for encoding the biosynthesis of various chemical classes of compounds such as PKSs, NRPSs, terpenoids, siderophores, lanthipeptides, and several other natural products. These compounds have been found to possess diverse biological activities and are known to synthesize a wide range of antibiotics with significant pharmaceutical potential [10,11]. Indeed, natural products and their structural analogues have played a crucial role in pharmacotherapy, especially in the treatment of cancer and infectious diseases [12]. However, the utilization of natural products in drug discovery has traditionally posed challenges due to various technical barriers. These challenges include the isolation, screening, optimization, and characterization of bioactive compounds. Recent high-tech and scientific developments, including improved analytical tools, genome mining, engineering methods, and advancements in culturable methods, have addressed some of these issues and created new possibilities to consider natural products as potential drug leads, particularly for combating AMR [13]. The screening of microbial natural products has gained significant attention in the search for novel therapeutic agents, particularly in combating AMR [14,15].

Forest ecosystems are characterized by their rich biological diversity of plants, animals, and microorganisms and their intricate interdependence [16,17]. Due to this complex ecology, potent microbial taxa in the forest soil such as actinomycetia are often difficult to isolate and culture, making discovery of novel antibiotics challenging [18,19]. To overcome this limitation and unlock the potential of uncultivable actinomycetia, researchers are actively working on enhancing cultivation methods. These efforts involve mimicking the natural environment of actinomycetia, optimizing growth media, and creating specialized culturing conditions to stimulate the growth and production of bioactive compounds [20]. Natural product discovery has recently shifted its attention to less-explored natural habitats in search of novel culturable strains producing new antimicrobial compounds [7,21,22]. Hence, the untouched Indo-Burma belt of northeast India with diverse microflora earns special importance in this regard [7]. Furthermore, the climatic conditions of the state of Assam with a diverse range of natural ecosystems, including forests, grasslands, and wetlands, serve as a suitable habitats for various microbial communities [23].

The present study aimed to explore the potential of actinomycetia in the PWS and DBWS as a source of new antimicrobial compounds to combat MDR pathogens. A total of 65 actinomycetia isolates were obtained from these forest ecosystems, and among them, 3 were identified as the most potent actinomycetia strains. The antimicrobial compounds produced by the most potent strains were characterized by GC-MS analysis. Additionally, the strains showed low MIC values, indicating their effectiveness against MDR pathogens. Furthermore, whole-genome sequencing of the three potent *Streptomyces* strains was performed to investigate their potential to produce diverse groups of smBGCs with therapeutic potential. This study proposes that these pristine forest ecosystems may contain novel actinomycetia species with significant antimicrobial properties, also earning special importance for further research into the antimicrobial potential of the diverse microflora.

## 2. Materials and Methods

### 2.1. Isolation of Pathogens from Urine Samples and In Vitro Antibiotic Susceptibility Testing

The urine samples of infected patients were collected from the pathological department of Guwahati Neurological Research Center (GNRC), Guwahati, Assam, India. They were labeled with the date of collection and sample code. The urine samples were transferred to the laboratory on the same day they were collected. In this study, UTI Hi-chrome agar (HiMedia, Mumbai, India) was used for the selective isolation and identification of pathogens from urine cultures. Synthetic chromogenic enzyme substrates are used in chromogenic media for the identification of pathogenic species based on their enzymatic action. Colorless substrates produce distinctive colors when cleaved by pathogen-specific enzymes [24]. All urine samples were inoculated aseptically onto UTI Hi-chrome agar media using a sterile loop. The culture plates were examined after 24 h of incubation at 37 °C. The isolated pathogens were then examined for antibiotic susceptibility assay. The antibiotics tested were nalidixic acid (30 µg), ampicillin (10 µg), levofloxacin (5 µg), co-trimoxazole (25 µg), norfloxacin (10 µg), ciprofloxacin (5 µg), cefazolin (30 µg), and nitrofurantoin (300 µg). The assay was performed using the HiMedia antibiotic sensitivity disk (HiMedia, Mumbai), as per the manufacture’s instruction. Antibiotic sensitivity was assayed from the diameter of the inhibition zones. As per the CLSI manual 2020 [25], based on the zone diameters, test clinical isolates were noted as either resistant (R), intermediate (I), or sensitive (S). The pathogens that showed resistance to most of the antibiotics were selected as test pathogens for in vitro bioassay.

### 2.2. Soil Sample Collection Site and Isolation of Actinomycetia from the Soil Samples

**Figure 1:**
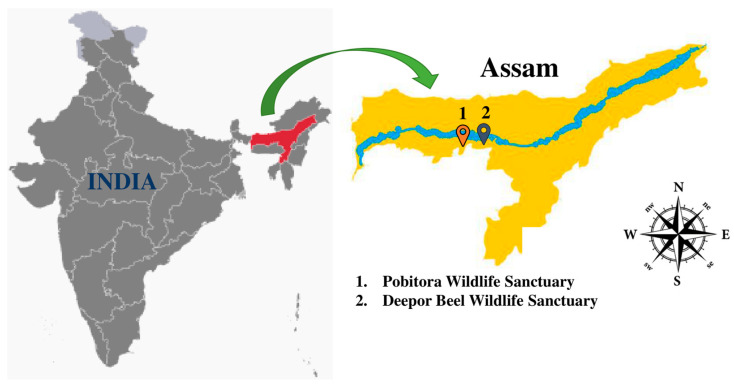
Soil sample collection site: soil samples were collected from two poorly explored forest ecosystems of Assam, India; (1) PWS. (2) DBWS.

Different locations of two protected forest ecosystems of Assam, PWS (26°12″–26°16″ N and 91°58″–92°05″ E) and DBWS (26.1177° N and 91.6494° E), situated in the northeastern part of India, were selected for the collection of soil samples (Figure 1). The soil is mainly composed of river alluvium, and the color of the soil ranges from reddish-brown to reddish-yellow. The soil is termed fertile clayey loam with silt. The rhizosphere soil samples were taken from the upper layer and 10 to 20 cm beneath the earth’s surface. The samples were collected in sterile zip lock bags and properly labeled with the date of collection and brought to the laboratory on the same day and stored at 4 °C until examined.

A serial dilution approach was used to isolate the actinomycetia from collected soil samples. A previously described, method [7] was followed to isolate actinomycetia from soil samples. A soil suspension from the collected soil sample was prepared with 5 g of soil in 100 mL of normal saline water (NaCl; 9 g/L), followed by incubation at 28 °C for 24 h with continuous shaking at 180 rpm. After allowing the mixtures to settle, sterile saline water was used to make dilutions up to 10^−4^, and then the dilutions were thoroughly mixed by vortex at maximum speed. For isolation, SA (Streptomyces Agar) and AIA (Actinomycetes Isolation Agar) media plates supplemented with amphotericin B (75 μg/mL) and rifampicin (2.5 μg/mL) were used. Amphotericin B and rifampicin were used against fungal and bacterial growth, respectively. Aliquots of 0.1 ml from each dilution were spread on the plates of isolation media and incubated at 28 °C and monitored after 48, 72, and 96 h. Streaking on GLM agar plate (Yeast extract, 3 g; malt extract, 3 g; peptone Type I, 5 g; starch, 10 g; agar, 20 g; distilled water, 1000 mL; pH 7.5) led to the purification of the actinomycetia isolate. The isolates were maintained in 20% glycerol at −80 °C. The morphology of the isolates was examined using scanning electron microscopy (SEM) [26].

### 2.3. Model Pathogens for In Vitro Antimicrobial Bioassay

Seven model pathogens were used for the in vitro antimicrobial bioassay: Gram-positive bacteria: (methicillin-resistant *Staphylococcus aureus*) MRSA (ATCC 43300); Gram-negative bacteria: *Klebsiella pneumoniae* (MTCC 3384), *Pseudomonas aeruginosa* (MTCC 741); yeast: *Candida albicans* (MTCC 227), and three MDR pathogens; *Enterococcus faecalis* (GNR7), *Pseudomonas aeruginosa* (GNR18), and *Escherichia coli* (GNR19). MTCC and ATCC strains were procured from Microbial Type Culture Collection, CSIR Institute of microbial technology cultures and HiMedia, Mumbai, respectively.

### 2.4. Evaluation of Actinomycetia against Model Pathogens and MDR Pathogens

A total of 65 actinomycetia isolates were selected for in vitro antimicrobial bioassay. Spot inoculation technique was used for the preliminary screening against different model pathogens on 5-day-old cultured GLM agar plate of actinomycetia isolates [7]. A clear zone was seen after 24–48 h of incubation at 37 °C and 28 °C for bacteria and yeasts, respectively. For secondary screening, the agar well diffusion method was followed [27]. The fermentation of the culture broth was performed by the method described previously [7]. A clear inhibition zone around the wells indicates that the test organism is sensitive to the corresponding broth. Each experiment was carried out thrice, and the mean value of the diameter of the inhibition zone (mm ± SE) was calculated.

### 2.5. Determination of Minimum Inhibitory Concentration (MIC)

The MIC of the ethyl acetate extract (EtAc) of three actinomycetia strains (EtAc-PBR1, EtAc-PBR36, and EtAc-DBR11) was examined by broth dilution method [7]. The concentration with no observable growth of the pathogen was noted as MIC.

### 2.6. Morphological Effect of Actinomycetia Isolate on Model Pathogen and MDR Pathogen

The antimicrobial effects of ethyl acetate extract of strain *Streptomyces* sp. PBR36 (EtAc-PBR36) on the cells of *Candida albicans* (MTCC 227) and *Escherichia coli* (GNR19) were investigated by SEM analysis [7].

### 2.7. Molecular Characterization

#### 2.7.1. Genomic DNA Extraction and PCR Amplification of 16S rRNA Gene

The actinomycetia isolates having the most potent antimicrobial activity against the model pathogens and MDR pathogens were selected for molecular identification. Nucleopore gDNA Fungal Bacteria Mini Kit (Genetix, New Delhi, India) was used for the extraction of DNA from the potent actinomycetia isolates. Universal primers, 27F (5′-AGA GTT TGA TCC TGG CTC AG-3′) and 1492R (5′-GGT TAC CTT GTT ACG ACT T-3′), were used for the amplification of 16S rRNA gene [28]. The reactions were executed in a Proflex PCR System (Applied Biosystems, Waltham, MA 02451, USA) in a total volume of 50 μL, consisting of 1.0 μL genomic DNA (10 ng), 0.2 μL of each primer (10 μM), 1×Taq DNA polymerase buffer (2.5 U), 0.2 mM of each dNTP, and 1 U Taq DNA polymerase enzyme. PCR was performed under the following conditions: initial denaturation at 94 °C for 5 min, followed by 35 cycles at 94 °C for 30 s, annealing at 52 °C for 30 s, 72 °C for 1 min, and a final extension at 72 °C for 7 min. Agarose gel (1.8% *w*/*v*) made in TAE buffer was used for the determination of PCR amplified products. A BioRad Gel Doc XR+ system (Hercules, CA, USA) was used for the visualization and imaging of the PCR bands. For molecular identification of the actinomycetia isolates, purified PCR products were sequenced by outsourcing to 1st BASE Laboratories, Malaysia.

#### 2.7.2. Molecular Identification and Phylogenetic Analysis

Phylogenetic analysis and calculation of pairwise sequence similarities of the actinomycetia isolates based on 16S ribosomal RNA gene were carried out using Nucleotide BLAST (Basic Local Alignment Search Tool) [29]. The partial 16S ribosomal RNA gene sequences were submitted to NCBI Gene Bank database. 16S rRNA sequences were aligned using MUSCLE and subjected to phylogenetic analysis by maximum likelihood method using MEGA X (windows ×64 version) [30] with 1000 bootstrap steps and Tamura-Nei distance model [31].

#### 2.7.3. Detection of Secondary Metabolite Synthesis Genes (NRPS and PKS-II)

PCR screening for PKS-II and NRPS biosynthetic genes was carried out using specific primers. PKS-II gene was amplified using degenerate primers KSαF (5′-TSG CST GCT TCG AYG GCS ATC-3′) and KSβR (3′-TCG CCG BAA GCC GCC NAA GGT-5′). NRPS genes were amplified using primers A3F (5′-GCS TAC SYS ATS TAC ACS TCS GG-3′) and A7R (5′-SAS GTC VCC SGT SCG GTA S-3′) [32]. PCR reactions were carried out in Proflex PCR System (Applied Biosystems, USA) in a final reaction volume of 50 µL consisting of template DNA (50 ng), 1×Taq DNA polymerase buffer, MgCl_2_ (1.5 mM), each dNTP (0.2 mM), 1 U Taq DNA polymerase enzyme, and each primer (0.2 µM). The amplified products for PKS-II and NRPS were evaluated in 1.8% (*w*/*v*) agarose gel made in 1×TAE buffer. The PCR bands were examined under UV light and documented using a BioRad Gel Doc XR+ system (Hercules, CA, USA).

### 2.8. Gas Chromatography–Mass Spectrometry (GC-MS) Analysis

GC-MS was performed to identify the chemical constituents present in EtAc-PBR1, EtAc-PBR36, and EtAc-DBR11 by the method described previously [7]. The peaks representing individual compounds were identified by matching the mass spectra with the National Institute of Standards and Technology (NIST), USA library.

### 2.9. Whole-Genome Sequencing Assembly and Annotation

Nucleopore gDNA Fungal Bacteria Mini Kit (Genetix) was used for the extraction of genomic DNA from the three most potent actinomycetia strains; *Streptomyces* sp. PBR1, *Streptomyces* sp. PBR36, and *Streptomyces* sp. DBR11. The genomic DNA library construction was performed using the Illumina Nextera DNA XT (Macrogen, Seoul, Republic of Korea). The paired-end illumina reads were quality checked using MultiQC, followed by adapter removal and trimming by Trimmomatic v 0.39 [33]. The genome was assembled using unicycler v 0.5.0 as a SPAdes-optimiser [34]. The assembly was then analyzed using quast v 5.2.0 [35]. The genome coverage and completeness were determined using BUSCO [36]. Furthermore, the genome assembly completeness was analyzed using the BUSCO tool [37]. The annotation of the genome and taxonomic identification was conducted using prokaryotic genome annotation pipeline (PGAP) and the average nucleotide identity (ANI) method [38,39]. The annotated contigs were processed in NCBI genomic workbench and submitted to NCBI using the genome submission wizard. The genome-wide identification, annotation, and analysis of smBGCs was performed using the antiSMASH (antibiotics and secondary metabolite analysis shell) tool (https://antismash.secondarymetabolites.org/ (accessed on 30 March 2022)) [40], and the results were compared with the MIBiG (Minimum Information about a Biosynthetic Gene cluster) gene cluster database. Rapid annotation subsystem technology (RAST), version 2.0 (https://rast.nmpdr.org/ (accessed on 5 April 2022)), was used to identify the virulence or antibiotic-resistant encoding genes [41].

### 2.10. Statistical Analysis

All experiments were performed in biological triplicate. The data were stated as the mean ± standard error of the mean. One-way ANOVA was performed in SPSS statistic 26.0 for the variation of inhibition among different actinomycetia strains with the 3 groups (3 MDR pathogens). A *p* < 0.001 was measured as significant. The test for variances within the strains in the antimicrobial assay is adjusted for all pairwise comparisons using the Bonferroni correction.

## 3. Results

### 3.1. Isolation and In Vitro Antibiotic Susceptibility Profile of MDR Pathogens

**Figure 2:**
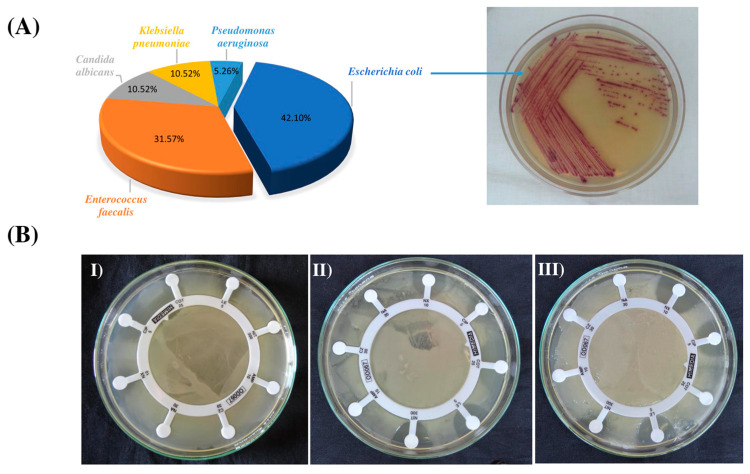
Isolation of pathogens from urine: (**A**) Most abundant pathogens isolated from culture plate, such as *Escherichia coli* (GNR19) on UTI Hi-chrome agar media. (**B**) In vitro screening of pathogens against antibiotics based on sensitivity disk (HiMedia), such as conducted for (I) *Escherichia coli* (GNR19); (II) *Enterococcus faecalis* (GNR7); and (III) *Pseudomonas aeruginosa* (GNR18).

A total of 28 cultures were examined from the urines of infected patients, and 19 pathogens were isolated. *E. coli* was found to be the most prevalent pathogen in the urine samples, accounting for 42.10% of the identified pathogens, followed by *Enterococcus faecalis* (31.57%), *Klebsiella pneumoniae* (10.52%), *Candida albicans* (10.52%), and *Pseudomonas aeruginosa* (5.26%) (Figure 2A). All of the isolated pathogens exhibited resistance to the tested antibiotics. The results of the antibiotic susceptibility profile are shown in Table S1. Out of nineteen isolated pathogens, seven showed resistance against all the antibiotics used in the antibiotic sensitivity studies and characterized as MDR isolates. Notably, five of the MDR isolates were *E. coli*. An increasing incidence of drug-resistant *E. coli* and *Enterococci* was observed. (Figure 2B).

### 3.2. Isolation and In Vitro Antimicrobial Bioassay of Actinomycetia against Model Pathogens and MDR Pathogens

**Figure 3:**
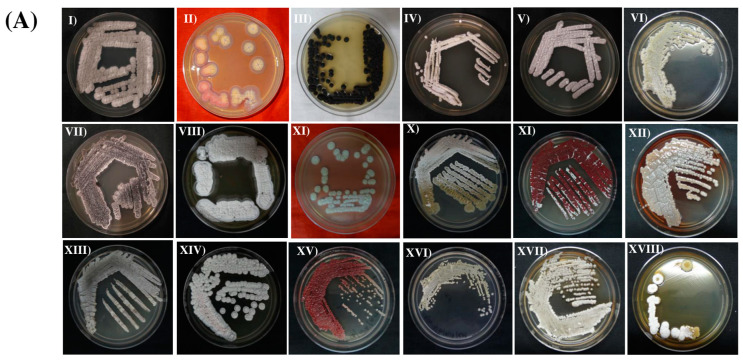

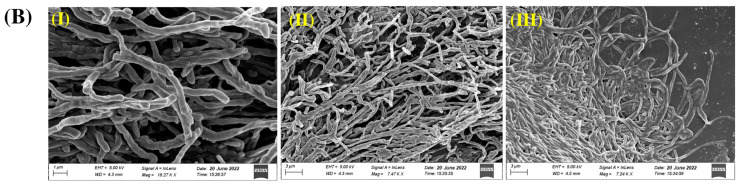
Isolation of actinomycetia from soil samples: (**A**) Pure culture plates of actinomycetia strains in GLM media. (I) *Streptomyces* sp. DBR11. (II) *Streptomyces* sp. PBR21. (III) *Actinomadura* sp. DBR17. (IV) *Streptomyces* sp. PBR30. (V) *Streptomyces* sp. DBR3. (VI) *Streptomyces* sp. PBR1. (VII); *Streptomyces* sp. DBR5. (VIII); *Streptomyces* sp. PBR11. (IX) *Streptomyces* sp. PBR19. (X) *Streptomyces* sp. PBR4. (XI) *Streptomyces* sp. DBR16. (XII) *Streptomyces* sp. PBR36. (XIII); *Streptomyces* sp. DBR1, (XIV); *Streptomyces* sp. PBR19, (XV) *Streptomyces* sp. DBR10. (XVI) *Nonomuraea* sp. DBR25. (XVII) *Streptomyces* sp. DBR4. (XVIII) *Streptomyces* sp. PBR35. (**B**) SEM image showing rectiflexibiles-type spore chains. (I) *Streptomyces* sp. DBR11. (II) *Streptomyces* sp. PBR1. (III) *Streptomyces* sp. PBR36.

The selective isolation process resulted in sixty-five morphologically different actinomycetia isolates from the soil samples of two poorly explored forest ecosystems. Thirty-nine actinomycetia were obtained from PWS and twenty-six from DBWS (Figure 3A). The SA media (*n* = 43) yielded more actinomycetia isolates, followed by AIA media (*n* = 22). The actinomycetia isolates were identified based on their morphological characters, such as colony morphology, color of substrate and aerial mycelium, color of diffusible pigments, and structure of hyphae (Figure 3B; Table S1).

**Figure 4:**
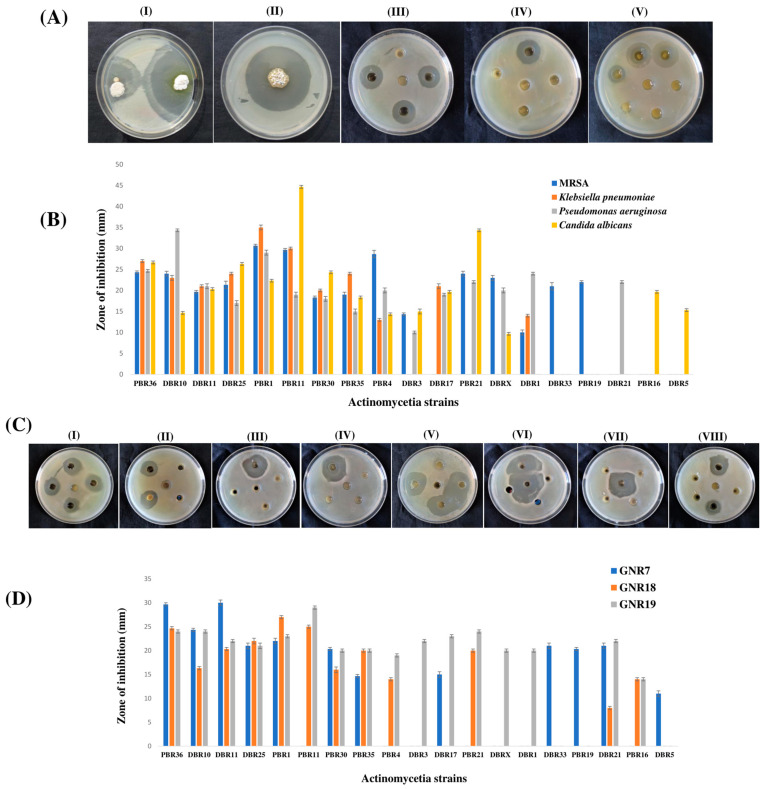
In vitro antimicrobial bioassay of actinomycetia extracts against model pathogens and MDR pathogens: (**A**) Antimicrobial activity by spot inoculation (I,II) and well diffusion method (III–V) against model pathogens: (I) *Candida albicans* (MTCC 227). (II,III) MRSA (ATCC 43300). (IV) *Klebsiella pneumonia* (MTCC 3384); (V) *Pseudomonas aeruginosa* (MTCC 741). (**B**) Bioactivity of fermentation broth of actinomycetia isolates against model pathogens, based on triplicate experiments. (**C**) Antimicrobial activity by well diffusion method against MDR pathogens: (I,II) *Pseudomonas aeruginosa*. (III–V) *Enterococcus faecalis*. (VI–VIII) *Escherichia coli*. (**D**) Bioactivity of fermentation broth of actinomycetia isolates against 3 MDR pathogens, based on triplicate experiments, against: GNR7; *Enterococcus faecalis*; GNR18; *Pseudomonas aeruginosa*; and GNR19; *Escherichia coli*.

Out of 65 actinomycetia isolates, 19 strains (29.23%) showed antimicrobial activity against at least 1 of the 4 model pathogens and 3 MDR pathogens. A total of 15 strains (78.9%) exhibited positive activity against MRSA (ATCC 43300), where Streptomyces sp. PBR1 exhibited a maximum zone of inhibition of 30.67 ± 0.33 mm. A total of 11 strains (73.33%) showed antimicrobial activity against *Klebsiella pneumoniae* (MTCC 3384), with the highest zone of inhibition being 35 ± 0.58 mm by *Streptomyces* sp. PBR1. The growth of *Pseudomonas aeruginosa* (MTCC 741) is inhibited by 15 actinomycetia strains (78.94%), with a maximum zone of inhibition of 34.33± 0.33 mm by *Streptomyces* sp. DBR10. Fifteen strains exhibited significant antifungal activity against *Candida albicans* (MTCC 227) with a maximum zone of inhibition of 47.00 ± 1.00 mm by *Streptomyces* sp. PBR11. Results of antimicrobial activity by spot inoculation and well diffusion method are shown in Figure 4. Furthermore, six actinomycetia strains, viz. *Streptomyces* sp. PBR36, *Streptomyces* sp. DBR10, *Streptomyces* sp. DBR11, *Nonomuraea* sp. DBR25, *Streptomyces* sp. PBR1, and *Streptomyces* sp. PBR35, can inhibit all the model pathogens, including MDR pathogens (Tables S3 and S4). The three most promising actinomycetia strains identified as *Streptomyces* sp. PBR1, *Streptomyces* sp. PBR36, and *Streptomyces* sp. DBR11 exhibited broad-spectrum antimicrobial activity, with the maximum inhibition against three MDR pathogens. The values that do not share the same superscript are significantly different at *p* < 0.001, as shown in Tables S3 and S4.

### 3.3. Minimum Inhibitory Concentration (MIC) Assay

MIC values of ethyl acetate extract (EtAc) of the three strains PBR1, PBR36, and DBR11 ranged from 50 to 0.097 µg/mL against three pathogens, namely MRSA, *Escherichia coli* GNR19 (clinical isolate), and *Candida albicans*, were determined by broth dilution technique. EtAc-PBR1 showed the lowest MIC of 0.195 µg/mL against MRSA, whereas the highest value was recorded against *Escherichia coli* GNR19 (3.125 µg/mL). EtAc-PBR36 showed the lowest MIC of 0.097 µg/mL against *Candida albicans*, whereas the highest was recorded against both MRSA (0.781 µg/mL) and *Escherichia coli* GNR19 (0.781 µg/mL). EtAc-DBR11 exhibited the lowest MIC of 0.195 µg/mL against *Candida albicans*, whereas the highest value was noted against *Escherichia coli* GNR19 (12.50 µg/mL) (Table 1). According to CLSI, 2020 recommendations for MIC of levofloxacin, *Escherichia coli* (GNR19) was found to be resistant to EtAc-DBR11 (MIC: 12.50 µg/mL), since ≤2 µg/mL was taken as susceptible, ≤4 µg/mL as intermediate, and ≥8 µg/mL as resistant. A 10% DMSO was used as a control and had no activity on the model pathogens.

**Figure 5:**
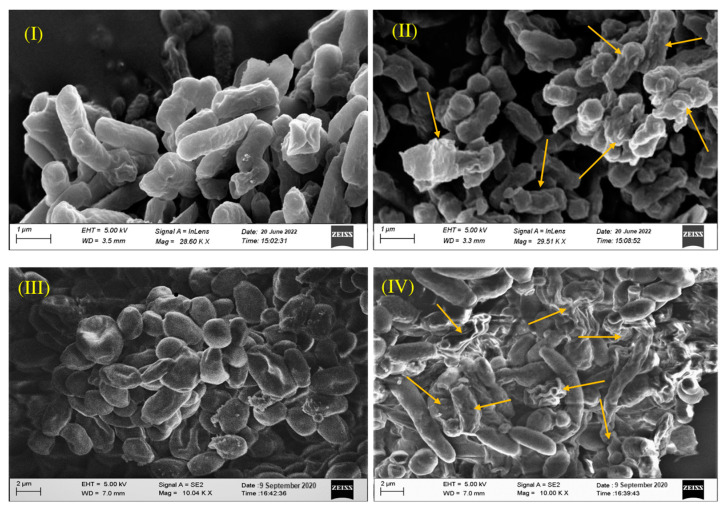
SEM image showing the effects of ethyl acetate extract of PBR36: (**I**,**III**) without treatment; (**II**,**IV**) treatment against *Candida albicans* MTCC 227 and *Escherichia coli* (GNR19), respectively, with 1 × MIC EtAc-PBR36. Arrow line denotes ruptured cells after treatment.

A considerable variation in the morphology was observed when the cells of MDR pathogen *Escherichia coli* (GNR19) and *Candida albicans* (MTCC 227) were treated with 1 × MIC EtAc-PBR36 (Figure 5). SEM analysis revealed that the treated cells were deformed and shrunk due to the loss of their cell integrity. However, actual cell morphology with intact cell surface was observed in the control cells.

### 3.4. Molecular Characterization and Phylogenetic Analysis of Actinomycetia Isolates Using 16S rRNA Sequences

16S ribosomal RNAs of 19 potent actinomycetia isolates were sequenced and submitted to the NCBI GenBank database with accession numbers MH922849-MH922864, MN069557, MH718314, and MK981152. The 16S rRNA gene sequences of the actinomycetia strains revealed sequence similarity percentage within 92–100% upon BLAST homology search in EzTaxon [https://www.ezbiocloud.net (accessed on 11 February 2021)]. Two actinomycetia strains, *Streptomyces* sp. PBR11 (GenBank accession no. MH718314, 1370 bp) and *Streptomyces* sp. PBR19 (GenBank accession no. MH922862, 1348 bp), exhibited very low sequence similarity to their closest *Streptomyces* sp. PBR11 showed 92.91% sequence similarity to their closest hit, while *Streptomyces* sp. PBR19 displayed a maximum sequence similarity of 93.96. Based on the partial 16S rRNA sequences, all 19 actinomycetia strains were taxonomically divided into 4 different genera. The genus *Streptomyces* (*n* = 16) was found to be dominant, followed by *Nocardia* (*n* = 1), *Actinomadura* (*n* = 1) and *Nonomuraea* (*n* = 1) (Table S5).

**Figure 6:**
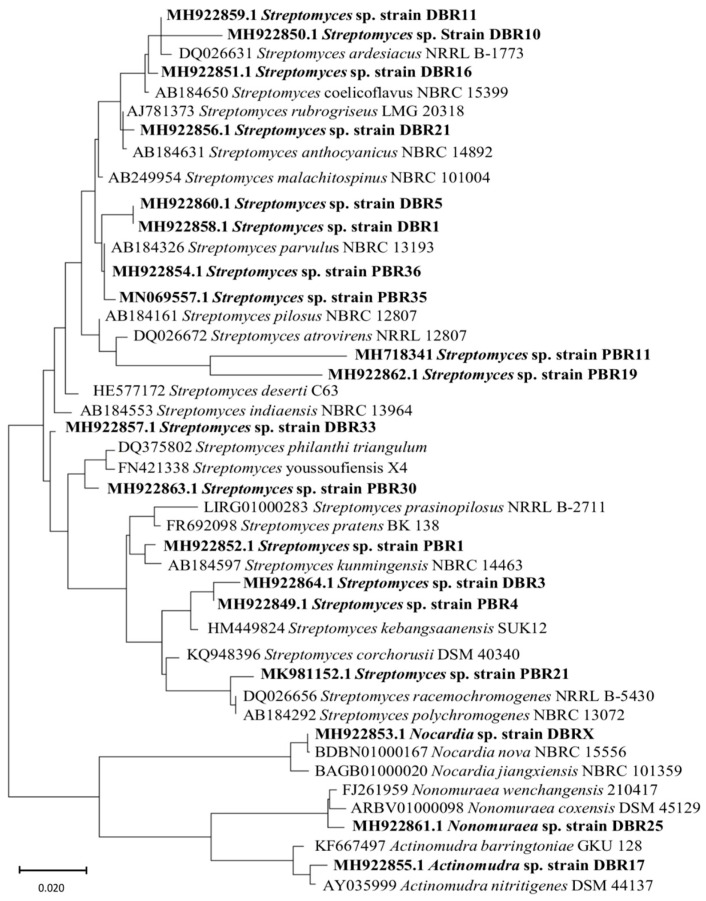
Phylogenetic tree of actinomycetia isolated from forest ecosystems and the closest type strains based on the 16S rRNA sequences: Sequences were aligned using MUSCLE and subjected to phylogenetic analysis by maximum likelihood method using MEGA X with 1000 bootstrap steps and Tamura–Nei distance model.

Maximum likelihood analysis of 16s rRNA gene sequences also revealed two major clusters, one with the subcluster containing *Streptomyces* species and another with rare actinomycetia, namely *Nocardia*, *Nonomuraea*, and *Actinomadura*. The strains PBR35, PBR11, PBR19, and PBR21 were distributed distinctly, indicating that they may be related to the distinct taxa within the genus *Streptomyces* (Figure 6).

### 3.5. Detection of Biosynthetic (PKS-II and NRPS) Genes

**Figure 7:**
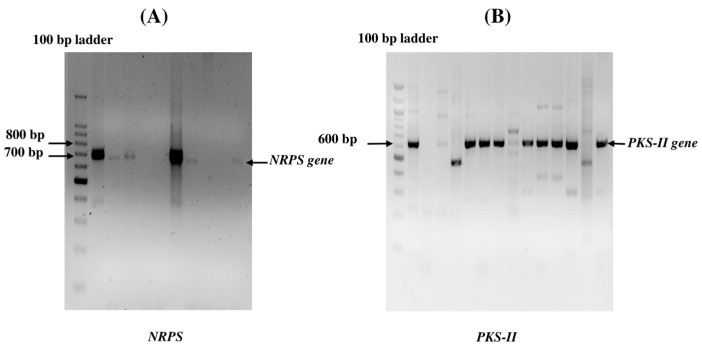
Agarose gel electrophoresis of PCR amplified products of actinobacteria: (**A**) Amplification of NRPS gene using A3F/A7R specific primers. (**B**) Amplification of PKS-II gene using degenerate primers KSαF/KSβR.

Nineteen potent actinomycetia isolates were assessed for the presence of different antimicrobial BGCs. Seventeen strains (89.47%) of actinomycetia showed an expected band size for the PKS type II gene, whereas the NRPS gene was detected in six strains (31.57%). Six actinomycetia strains, viz. *Streptomyces* sp. PBR30, *Streptomyces* sp. PBR35, *Nocardia* sp. DBRX, *Streptomyces* sp. PBR1, *Streptomyces* sp. PBR36, and *Streptomyces* sp. DBR11, showed positive results for both BGCs, i.e., PKS-II and NRPS (Figure 7).

### 3.6. Gas Chromatography–Mass Spectrometry Analysis

The chemical profiling of the three most potent actinomycetia strains was performed using GC-MS analysis. A total of 19 major chemical compounds were detected in the ethyl acetate extracts of the 3 strains based on their molecular weight and retention time by comparing their mass spectra with the NIST database. In the ethyl acetate extract of *Streptomyces* sp. PBR1, i.e., EtAc-PBR1, nine major chemical compounds were detected, namely 5-amino-6-methoxy-8-nitroquinoline, isonipecotic acid, N-(3-fluoro-5-trifluoromethylbenzoyl)-, eicosyl ester, acetic acid, ydrazine-, ethyl ester, 4-bromo-2-trifluoromethylphenyl isothiocyanate, haloperidol, TMS derivative, spiro [5.5] undecane, 1-methylene, 9-acridanone, 1-ethoxy-3-methoxy-, 10-methyl-, 1-anthracenyl-2-pyridyl ketone, and fluoxymesterone. In EtAc-PBR36, four chemical compounds, resibufogenin, o-veratramide, 5-dicyanomethylene-9-diethylamino-6-fluorobenzo[a] phenoxazine and lanostan-3. β. -ol, 11. beta. 19-epoxy, were identified. Moreover, six major compounds, 2-(4-bromo-phenyl)-indolizine, 2-bromo-N-[4-bromo-2-(2-bromo-benzoyl)-phenyl]-acetamide, D: A-friedooleanan-3-ol, (3. α.), cyclopentanecarboxylic acid, 1-(4-ethoxy-4-oxo-2-butenyl)-2-oxo-, ethyl ester, L-Cysteine, N-acetyl-, methyl ester, acetate, and desflurane, were detected in the EtAc-DBR11 strain. Most of the detected compounds were reported to exhibit anticancer, antimicrobial, antiviral, antimalarial anti-inflammatory, and antituberculosis activities (Table 2A–C). The chemical structures of the identified compounds are shown in Figure S1.

### 3.7. Genome Structure of Three Actinomycetia Strains

Genomic libraries of the three most potent actinomycetia strains were constructed using a Nextera XT DNA library preparation kit (Illumina Inc., Macrogen, Seoul, Republic of Korea). The genomic DNA was sequenced by the illumina HiSeq platform, using paired-end reads (150 bp). The de novo assembly of the sequences was annotated by prokaryotic genome annotation pipeline (PGAP). The assembled genome size of *Streptomyces* sp. DBR11 was 8,076,963 bp with G + C content of 72.09%. In the case of *Streptomyces* sp. PBR1, the assembled genome size was 9,557,226 bp with G + C content of 71.75%, and the complete genome of *Streptomyces* sp. PBR36 was found to be composed of 7,334,189 bp with G + C content of 72.82%. The complete genome sequences of the three actinomycetia strains were deposited at NCBI under WGS accession numbers JAMOLN000000000, JAMOLM000000000, and JAMOLO000000000 and BioProject number PRJNA834923. The WGS accessions, assembly statistics, and genome coverage are listed in Table S6.

### 3.8. Identification of Biosynthetic Gene Clusters and Antibiotic Resistant Genes

The secondary metabolites of three actinomycetia strains, *Streptomyces* sp. DBR11, *Streptomyces* sp. PBR1, and *Streptomyces* sp. PBR36, were predicted by employing antiSMASH (version 6.0.1) [40]. Nucleotide sequence analysis of all three actinomycetia strains revealed a diverse group of BGCs responsible for the production of bioactive secondary metabolites. They were involved in the biosynthesis of different classes of gene clusters, such as PKSs, NRPS, terpene, lipopeptide, lanthipeptide, siderophore, butyrolactone, NAPAA (nonalpha polyamino group acids), saccharides, and hybrid gene clusters of NRPS-PKS and Terpene + PKS. Nucleotide sequence analysis of *Streptomyces* sp. DBR11 revealed 31 smBGCs responsible for secondary metabolite production, out of which 24 are related to known types of smBGCs, such as antibiotics, bioactive compounds, and other products. Eight smBGCs were associated with the synthesis of known antibiotics: carbapenem MM4550, albaflavenone, borrelidin, hormaomycin A1-A6, niphimycins C-E, enduracidin, stenothricin, and icosalide A. Ten smBGCs displayed 100% homology to known secondary metabolites: albaflavenone, icosalide A, ashimides, ectoine, rhizomide A-C, isorenieratene, coelibactin, germicidin, sapB, and hopene. Four smBGCs revealed high to moderate homology (50–85% of genes showed similarity) to known gene clusters of undecylprodigiosin, desferrioxamin B/desferrioxamine E, melanin, and spore pigment. Ten BGCs exhibited low homology (<30% of genes showed similarity), and six other BGCs showed no homology to any known secondary metabolites.

*Streptomyces* sp. PBR1 contains 33 BGCs in its genome, 26 of which were involved in the biosynthesis of known secondary metabolites. Among them, 12 gene clusters were annotated to known antibiotics, such as lankamycin, streptovaricin, lankacidin C, stenothricin, chaxamycin A-D, glycinocin A, albaflavenone, murayaquinone, granaticin, ficellomycin, aurantimycin A, merochlorin A-D/deschloro-merochlorin, and A–B/isochloro-merochlorin B/dichloro-merochlorin B. Moreover, 11 antibiotics were associated with PKS/NRPS pathways, whereas 2 of them were annotated by hybrid (NRP+ Polyketide) and 1 by (Terpene + Polyketide: Type III) gene clusters. Four smBGCs displayed 100% homology to the known gene clusters of albaflavenone, ectoine, and keywimysin, and 2-methylisoborneol and 8 smBGCs represented high to moderate homology (50–85% of genes showed homology) with the known gene clusters of lankamycin, streptovaricin, albaflavenone, desferrioxamin B/desferrioxamine E, 4-Z-annimycin, 4-hexadecanoyl-3-hydroxy-2-(hydroxymethyl)-2H-furan-5-one, flaviolin, and hopene. Fifteen BGCs represented low homology (<40% of genes showed similarity) and six gene clusters showed no homology to any known secondary metabolites.

*Streptomyces* sp. PBR36 contained 29 smBGCs, 26 of which are known to be associated with secondary metabolites. Significantly, 11 gene clusters encode known antibiotics, albaflavenone, stenothricin, actinomycin D, abyssomicin C, ulleungmycin, streptovaricin, griseoviridin, tylactone, aurantimycin A, and istamycin, where two of them were annotated by hybrid (NRP+ Polyketide and NRP: Cyclic depsipeptide + Polyketide: Trans-AT type I) gene clusters. Six smBGCs revealed 100% similarity to some secondary metabolites, such as albaflavenone, ectoine, sapB, hopene, germicidin, and isorenieratene. Four smBGCs displayed moderate to high homology (60–90% of genes showed homology) to the known gene clusters of actinomycin D, desferrioxamin B, desferrioxamine E, spore pigment, and coelichelin. In total, 16 BGCs revealed low homology (<42% of genes showed similarity), and 3 BGCs showed no homology to any known secondary metabolites.

**Figure 8:**
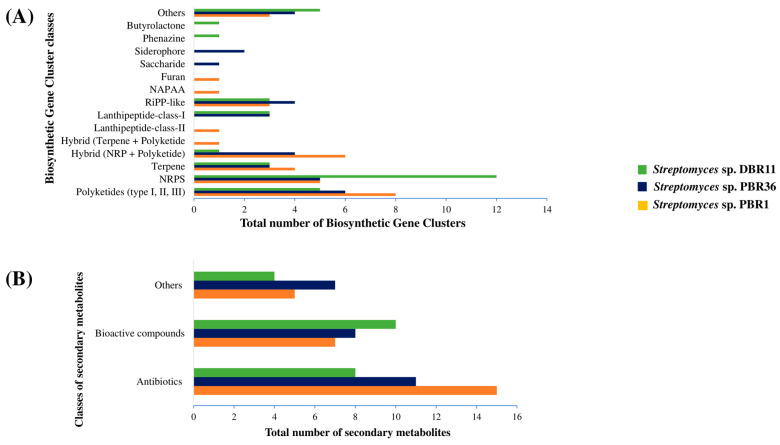

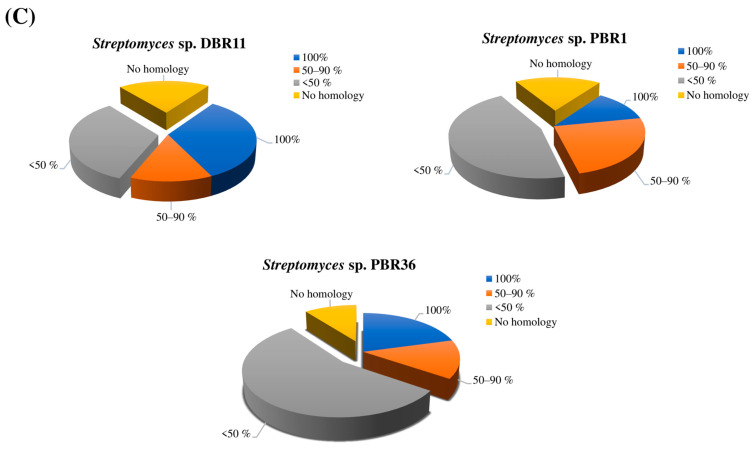
Genome mining for identifying smBGCs: (**A**) Distribution of antiSMASH hits of major classes of smBGCs identified in the genome of three actinomycetia strains; *Streptomyces* sp. DBR11, *Streptomyces* sp. PBR1, and *Streptomyces* sp. PBR36. The clusters of the “Hybrid” type that were predicted to belong to more than one type of BGCs were combined. BGCs predicted to belong to the hopene, ectoine, germicidin, sapB, melanin, geosmin, spore pigment keywimysin, carotenoid, 2-methylisoborneol, or flaviolin types were grouped under “Other”. (**B**) Comparison of the number and types of secondary metabolites found in the genomes of the three actinomycetia strains. (**C**) Similarity percentage of BGCs identified in the genome of three actinomycetia strains to known homologous gene clusters. No homology shown by the gene clusters revealed no similarity shared by the BGCs to known gene clusters.

The presence of highly similar gene clusters in all the three actinomycetia strains suggested they have tremendous potential to generate these secondary metabolites. Interestingly, most of the gene clusters had low (<50%) or no similarity to other known smBGCs, suggesting that the three strains could be a source of novel antibiotics. Complete genome mining results are shown in Figure 8, Table 3A–C.

**Figure 9:**
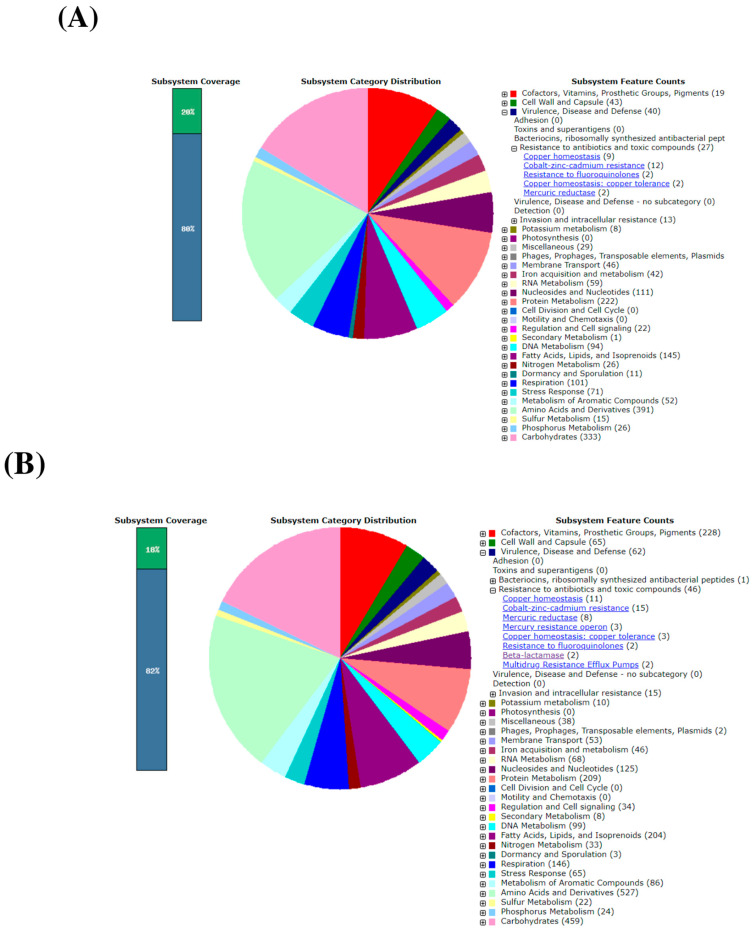

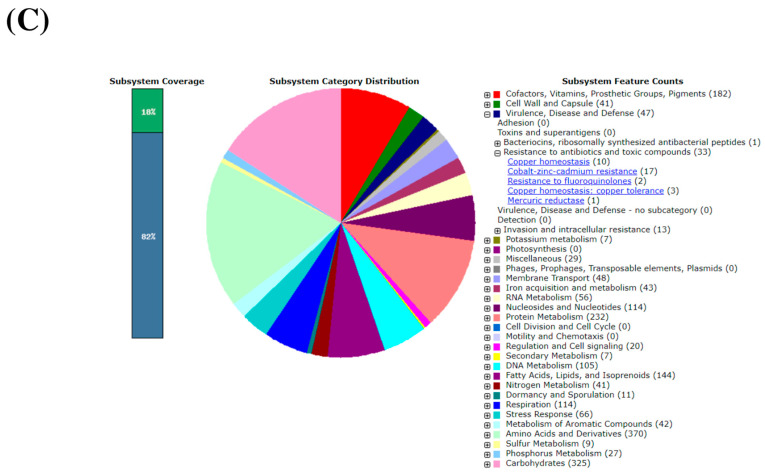
An overview of the subsystem category of the annotated whole genomes of three actinomycetia strains showing virulence or antibiotic resistance encoding genes using RAST pipeline: (**A**) *Streptomyces* sp. PBR36. (**B**) *Streptomyces* sp. PBR36. (**C**) *Streptomyces* sp. DBR11. Green color in the color bar represents features that are found in the RAST subsystem. The blue color represents features not assigned to a subsystem.

According to the RAST annotation of the draft genomes of all three actinomycetia strains, PBR36, PBR1, and DBR11 contain the highest number of coding sequences (391, 527, and 370 counts, respectively) for the subsystem of amino acids and derivatives, followed by the subsystem of carbohydrates (333, 459, and 325 counts, respectively) and protein metabolism (222, 209, and 232 counts, respectively). No virulence or toxin-encoding genes were found in the genomes of the three strains. Fluoroquinolone-resistant genes were found in conserved domains of all three actinomycetia strains, whereas genes encoding beta-lactamase resistance were found only in *Streptomyces* sp. PBR1 strain (Figure 9)

## 4. Discussion

AMR is a major public health concern, particularly for common bacterial infections such as UTIs, sepsis, sexually transmitted infections, and some forms of diarrhea [58]. Furthermore, the development of new antibiotics has slowed down significantly in recent decades, leading to a limited pipeline of effective drugs to combat resistant bacterial pathogens.

In the present study, the potential of forest-derived actinomycetia was investigated against MDR pathogens. A total of 19 pathogens were isolated from the urine samples obtained from infected patients. The findings showed that *Escherichia coli* (42%) was the most frequently occurring pathogen in the urine samples, followed by *Enterococcus faecalis* (32%), *Klebsiella pneumoniae* (11%), *Candida albicans* (11%), and *Pseudomonas aeruginosa* (5%). In vitro antibiotic sensitivity study was performed to investigate the susceptibility of the pathogens to eight different standard antibiotics. The study indicates that only 11% of the pathogens were sensitive to both ciprofloxacin (5 µg) and nalidixic acid (30 µg), while 16% of the pathogens were sensitive to ampicillin (10 µg). This suggests that the tested pathogens are resistant to these antibiotics. Moreover, 21% of the pathogens were sensitive to levofloxacin (5 µg), while co-trimoxazole (25 µg) and norfloxacin (10 µg) were effective against 32% and 31% of the pathogens, respectively. According to a recent systematic review analysis, Gram-negative bacteria have high resistance rates to co-trimoxazole in certain countries in the Asia-Pacific region: India (64–74%), Bangladesh (58%), and Bhutan (53%) [59]. Drugs like ciprofloxacin, which belong to the class of antibiotics known as fluoroquinolones, were once commonly prescribed for UTIs. However, physicians are now more cautious in prescribing ciprofloxacin in areas where the resistance rates are high. A previous study also demonstrated that ciprofloxacin (500 mg) is recommended twice daily for 10–14 days for only mild UTIs [60]. In comparison with the antibiotics listed above, cefazolin (30 g) 36.84% and nitrofurantoin (300 g) 36.84% may be better drug options, because they were effective against pathogens and also showed a clear zone in the antibiotic sensitivity test. According to Antibiotic Research, UK [61] and UNEP (United Nations Environment Program) [62], AMR is a global health concern, and currently, it causes approximately 700,000 deaths annually worldwide. If appropriate measures are not taken to address the issue, it is projected that the annual death toll could reach a staggering 10 million by 2050. Based on in vitro antibiotic susceptibility assay, seven MDR pathogens were isolated from the urine sample of infected patients. Three bacterial pathogens, viz. GNR7 (*Enterococcus faecalis*), GNR18 (*Pseudomonas aeruginosa*), and GNR19 (*Escherichia coli*), were considered to be potent MDR pathogens, because they were resistant to all of the antibiotics used in the antibiotic susceptibility test. They were therefore considered as test pathogens for further in vitro bioassay against isolated actinomycetia strains.

The plant rhizosphere is a hotspot for microbial interactions and activities and is widely being studied by scientists [63,64]. Actinomycetia are important soil microbes that dominate the rhizosphere and contribute to various ecological processes. However, despite their significance, only a small portion actinomycetia has been studied [65]. Assam, located in one of the world’s mega-biodiversity hotspots, is indeed home to diverse forest ecosystems that are considered poorly explored [7]. These forests of Assam harbor a wide range of microbial communities and are recognized as important resources of actinomycetia [7,66]. In the present study, a total of 65 morphologically diverse actinomycetia were isolated from two less explored forest ecosystems of Assam, namely PWS and DBWS. The morphological characterization of the 65 actinomycete isolates was assessed by analyzing various visible morphological characteristics. These characteristics included colony morphology, aerial and substrate mycelium color, and the presence of diffusible pigments. Thirteen isolates of *Streptomyces* produced a diffusible pigment, which is considered a key feature for the morphological characterization of the *Streptomyces* [67]. In addition to pigment production, other characteristics such as spore chain length, hyphae structure, and fragmentation of mycelium are also considered for the classification of actinomycetia [68]. The SEM analysis revealed that the aerial mycelia of *Streptomyces* sp. DBR11, *Streptomyces* sp. PBR1, and *Streptomyces* sp. PBR36 are long, straight, and branched with chains of conidiospores. The mycelium of all three actinomycetia is closed, compact, and rectiflexibiles type (Figure 3B).

Actinomycetia are considered one of the most prolific sources of bioactive compounds, and their metabolites have been extensively studied for their potential applications in medicine [21,69]. The present study investigated 65 actinomycetia isolates, and among them, 19 were identified as potent actinomycetia with significant antimicrobial activity on Gram-positive bacteria, Gram-negative bacteria, *Candida species*, and 3 MDR pathogens. During the screening of actinomycetia isolates for antagonistic activity, seven isolates exhibited broad-spectrum activity against all the pathogens, and three strains, viz. *Streptomyces* sp. PBR1, *Streptomyces* sp. PBR36, and *Streptomyces* sp. DBR11, exhibited broad-spectrum activity with a significantly larger zone of inhibition compared with the other isolates. Both PBR1 and PBR36 were isolated from soil samples of PWS, while DBR11 was isolated from DBWS. Similarly, in our recent work with *Streptomyces* sp. PBR11 isolated from a forest ecosystem, we observed similar broad-spectrum antimicrobial activity and characterized the presence of an antituberculosis drug ethambutol. In addition, the study demonstrated antimicrobial activity of its bioactive fraction against 19 test pathogens, including MDR clinical pathogens and dermatophytes [7]. Likewise, the present study also suggests that forest ecosystems are an excellent resource of many actinomycetia exhibiting antagonistic activity against different drug-resistant pathogens. Currently, no studies have been published on the investigation of the antimicrobial activity of actinomycetia from the ecosystem of DBWS of Assam. As a Ramsar site on the southwest outskirts of Guwahati, Assam, DBWS merits special attention for the investigation of novel actinomycetia with therapeutic applications. A Ramsar site is a wetland area that has been given international significance under the Ramsar Convention, also known as “The Convention on Wetlands”, an international environmental agreement that was established on February 2nd, 1971, in Ramsar, Iran by UNESCO [70]. This is the first report on the scientific evidence of actinomycetia from DBWS that demonstrated broad-spectrum activity against MDR clinical pathogens.

Crude ethyl acetate extracts of three actinomycetia strains, *Streptomyces* sp. PBR1, *Streptomyces* sp. PBR36, and *Streptomyces* sp. DBR11, were selected for MIC studies based on their antimicrobial potential against the MDR pathogens. *Streptomyces* sp. PBR1 exhibited the lowest MIC against MRSA (0.195 µg/mL). *Streptomyces* sp. PBR36 exhibited the lowest MIC against *Candida albicans* (0.097 µg/mL) and exhibited an MIC of 0.781 µg/mL against both MDR *Escherichia coli* (clinical isolate, GNR19) and MRSA. *Streptomyces* sp. DBR11 exhibited the lowest MIC against *Candida albicans* (0.195 µg/mL). Recently, similar findings were reported, where EA-Kz-24 exhibited the lowest MIC against MRSA ATCC 43,300 and *Candida albicans* MTCC 227 (0.024 µg/mL) [48]. Notably, the MIC values showed by the crude extract of the three potent stains were significantly lower than those of the standard antimicrobial drugs. This is the first report where the actinomycetia isolates showed broad-spectrum activity against MDR pathogens at the lowest MIC values. Furthermore, SEM analysis revealed remarkable morphological and physiological changes, such as cell shrinkage and ruptured cell walls in the treated cells of *Candida albicans* MTCC 227 and *Escherichia coli* GNR19. This suggests that the antimicrobial action of EtAc-PBR36 against pathogens is mediated by a membrane disruption mechanism that inhibits further cell growth.

16S rRNA gene sequencing has been widely used as an effective molecular technique for genus-level classification of bacteria [71]. Nineteen antagonistic actinomycetia isolates were partially identified by 16S rRNA gene sequencing. Nucleotide sequences of the isolates showed 92–100% similarity with the reference sequence in the EzTaxon database. Two strains, *Streptomyces* sp. PBR11 (92.91%) and *Streptomyces* sp. PBR19 (93.96%), presented very low sequence similarities to their nearest type strain and formed a distinct subclade from the other members of actinomycetia strains, which suggested that these two strains can be considered under the novel taxa. The three most potent actinomycetia strains, PBR1, PBR36, and DBR11, were identified as *Streptomyces* sp. Based on the similarity search of 16S rRNA gene sequences. However, upon taxonomic identification by ANI method [39] from WGS, the isolates were identified as *Streptomyces coelicoflavus*, *Streptomyces longispororuber*, and *Streptomyces parvulus*, respectively, with high confidence levels. Phylogenetic analysis by 16s rRNA gene sequences revealed two major clusters, the first cluster mainly comprises *Streptomyces* species, and in the other, rare actinomycetia such as *Nocardia*, *Nonomuraea*, and *Actinomadura*. The genetic diversity in the present study suggests that the soil of protected forest ecosystems of Assam is a rich source of *Streptomyces*, indicating that these forest areas could be a storehouse of many novel and rare actinomycetia with therapeutic potentials.

The metabolic gene clusters PKS-II and NRPS play key roles in the biosynthesis of bioactive secondary metabolites, which are responsible for the production of therapeutically relevant natural products, such as antibiotic, antifungal, antiviral, antitumor, and anticancer compounds [72]. Out of the 19 potent actinomycetia strains, 17 strains (89.47%) of actinomycetia exhibited positive results for PKS-II gene, whereas the NRPS gene was detected in 6 actinomycetia strains (31.57%). Both biosynthetic genes, PKS-II and NRPS, were detected in six strains viz. *Streptomyces* sp. PBR30, *Streptomyces* sp. PBR35, *Nocardia* sp. DBRX, *Streptomyces* sp. PBR1, *Streptomyces* sp. PBR36, and *Streptomyces* sp. DBR11. Notably, most of the potent strains, especially *Streptomyces* sp., were found to contain PKS-II genes, which might be responsible for their involvement in the biosynthesis of antimicrobial secondary metabolites. These findings also corroborate the previous studies conducted by other researchers [21,73]. The lack of amplification of PKS-II and NRPS gene sequences in some of the potent actinomycetia strains may be due to the absence of these specific biosynthetic genes in their genome, and secondary metabolites may be produced through some other biosynthetic pathways [21].

GC-MS is an effective analytical tool that is often used to identify metabolites from different sources by their MS fingerprint [74,75]. Several research articles have been published on GC-MS-based research for natural product chemistry and drug discovery [7,21]. In the present study, solvent extracts from three actinomycetia strains, *Streptomyces* sp. PBR1, *Streptomyces* sp. PBR36, and *Streptomyces* sp. DBR11, were subjected to GC-MS analysis to study the bioactive compounds produced by them. A total of nineteen major chemical compounds were detected based on their retention time and abundance, of which twelve are known to exhibit biological activities. For instance, isonipecotic acid, N-(3-fluoro-5-trifluoromethylbenzoyl)-, and eicosyl ester detected in the ethyl acetate extract of *Streptomyces* sp. PBR1 have potential anticonvulsant properties [42]. 4-bromo-2-trifluoromethylphenyl isothiocyanate exhibited antiviral activity against *Zika* virus [43]. An organofluoride compound, haloperidol TMS derivative, acts as a novel potent calcium channel blocker with vasodilator activity [44]. Moreover, three other compounds detected in the ethyl acetate extract of PBR1 strain, viz. Spiro [5.5] undecane, 1-methylene, 9-acridanone, 9-Acridanone, 1-ethoxy-3-methoxy- 10-methyl, and fluoxymesterone detected in the ethyl acetate extract of PBR1 strain, exhibited antimicrobial, antiviral, antimalarial, anti-inflammatory, and antianemia activities [45,46,47,48,49]. Similarly, in *Streptomyces* sp. PBR36, four bioactive compounds, resibufogenin, o-veratramide, 5-dicyanomethylene-9-diethylamino-6-fluorobenzo[a] phenoxazine, and lanostan-3. β. -ol, 11. beta,19-epoxy, have been identified to have anticancer, antimicrobial, antitumor, antimalaria, anti-*M. tuberculosis*, and anti-inflammation activities, respectively. The results are also found to be in agreement with previous studies [50,51,52,53,54,55]. Two compounds, L-cysteine, N-acetyl-, methyl ester, acetate, and desflurane, detected in *Streptomyces* sp. DBR11, exhibited mucolytic, antioxidant [76], and anesthetic activity [57], respectively. However, reports on the biological activities of compounds, such as 5-amino-6-methoxy-8-nitroquinoline, acetic acid, hydrazino-, ethyl ester, 1-anthracenyl-2-pyridyl ketone, 2-(4-bromo-phenyl)-indolizine, 2-Bromo-N- (4-bromo-2-(2, bromo-benzoyl)-phenyl)-acetamide, D: A-friedooleanan-3-ol, (3. α), and cyclopentanecarboxylic ac-id,1-(4-ethoxy-4-oxo-2-butenyl)-2-oxo-, ethyl ester, have not been published.

Genome mining is becoming a popular approach for accelerating the identification and characterization of the diverse classes of smBGCs [76,77]. The genome mining of the three most potent actinomycetia strains, *Streptomyces* sp. DBR11, *Streptomyces* sp. PBR1, and *Streptomyces* sp. PBR36 has revealed the presence of approximately 29–33 BGCs associated with the biosynthesis of PKSs (type I, II, and III PKSs), NRPSs, terpene, lipopeptide, lanthipeptide, siderophore and butyrolactone, NAPAA (nonalpha polyamino group acids), saccharides, and other natural products. The presence of such a diverse array of BGCs indicates that all three strains have the potential to produce a wide range of secondary metabolites. Lanthipeptides, like PKSs and NRPSs, are also biologically significant chemical classes involved in the production of antimicrobial drugs. For example, cadasides, malacidins, and teixobactin are all peptide antibiotics that have been reported in recent years for their promising antimicrobial activity against various MDR bacterial pathogens [78,79,80,81]. *Streptomyces* sp. DBR11 and *Streptomyces* sp. PBR36 were found to have lanthipeptide class-I gene clusters, while the PBR1 strain had lanthipeptide class-II gene clusters. Also, most of the BGCs identified in these three strains are assigned to the polyketide domain, followed by NRPS and terpene biosynthesis, while in the genome of *Streptomyces* sp. DBR11, most of the BGCs encode the biosynthesis of NRPS, followed by PKSs, lanthipeptides, and terpenes. One of the key findings of this study is that actinomycetia strains isolated from the same area have similar sets of BGCs. This similarity suggests that these strains may have acquired the genes responsible for the biosynthesis of these secondary metabolites through horizontal gene transfer (HGT) [82]. Furthermore, antiSMASH analysis of three actinomycetia strain genomes revealed that the predicted gene clusters in these strains have no significant similarity or homology with known secondary metabolites. The lack of similarity suggests that the bioactive metabolites produced by these strains might be novel. Researchers are particularly interested in hybrid systems of BGCs, because high levels of secondary metabolites can be derived from a combination of NRPS and PKS gene clusters, resulting in a wide range of structures and diverse chemical compounds with high therapeutic potential [83]. Remarkably, all three *Streptomyces* strains carried NRPS-PKS and Terpene + PKS hybrid gene clusters.

It has previously been established that strains of the same bacterial species frequently encode a core set of BGCs, but they can also encode some additional clusters that differ significantly between strains [84]. *Streptomyces* sp. PBR1 and *Streptomyces* sp. PBR36 both contain smBGC, which codes for streptovaricin, a macrolide antibiotic with exceptional antimicrobial activity against MRSA and *Mycobacterium tuberculosis* [85]. Likewise, the strains also carried a gene cluster for aurantimycin A, a depsipeptide antibiotic with potent antitumor and antimicrobial activity [86]. BGCs coding for stenothricin, a nonribosomal peptide-derived antibiotic, and albaflavenone, a sesquiterpene antibiotic, was found in the genomes of all three actinomycetia stains. Stenothricin exhibited strong antibacterial activity [87], and albaflavenone has shown cytotoxic activity [88]. Moreover, other gene clusters which were detected in *Streptomyces* sp. PBR1 were responsible for production of lankamycin [89], lankacidin C [90], chaxamycin A-C [91], glycinocin A [92], murayaquinone [93], ficellomycin [94], and merochlorin A [95]. All of these smBGCs demonstrated significant antimicrobial activity. Gene clusters for other secondary metabolites like granaticin and violapyrone B were also detected in the genome of PBR1 stain. Granaticin is known to exhibit antibacterial and anticancer activities [96], and violapyrone B showed antibacterial, antitumor [97], and anti-influenza A (H1N1) activity [98].

Similarly, *Streptomyces* sp. PBR36 contains smBGCs that encode for ulleungmycin [99], griseoviridin [100], tylactone [101], and istamycin [102], all of which have antimicrobial activity against MDR bacterial pathogens. PBR36 also encodes the biosynthesis of abyssomicin C and actinomycin D gene clusters. Abyssomicin C is evidenced to possess significant antibacterial activity against Gram-positive bacteria, including MDR and vancomycin-resistant bacterial strains [103]. Likewise, actinomycin D is a well-known antibiotic used to treat various cancers [104,105]. *Streptomyces* sp. DBR11 contains smBGCs, which encode carbapenem MM4550 [106], borrelidin [107], hormaomycin A1-A6 [108], niphimycins C-E [109], enduracidin [110,111], and icosalide A [112] antibiotics with potent antimicrobial activity against various bacterial pathogens including MDR pathogens.

Gene clusters for bioactive compounds, such as desferrioxamin B/desferrioxamine E, foxicin A, polyoxypeptin, ashimides, herboxidiene, undecylprodigiosin, and chejuenolide A-B, were detected in the three potent actinomycetia strains. Desferrioxamine, as an iron-chelating molecule, is frequently used in clinical conditions to treat iron overload and for the treatment of hereditary hemochromatosis and thalassemia [113]. Similarly, foxicin showed moderate antibiotic activity in a recent study [114]. Polyoxypeptin is a potent apoptosis inducer [115], ashimides showed cytotoxic activity [116], herboxidiene is a potent antitumor agent [117], undecylprodigiosin is reported to exhibit antimicrobial, anticancer, and antimalarial activities [118], and chejuenolide A-B exhibited antimicrobial activity [119]. The synthesis of these bioactive secondary metabolites may be responsible for the broad-spectrum antimicrobial activity of the three potent actinomycetia strains against MDR pathogens.

Many clinically significant antibiotics are produced by actinomycetia, particularly from the genus *Streptomyces*. BGCs often contain genes that offer resistance to the antibiotics they produce. These antibiotic-resistant genes play a vital role in the self-defense mechanism against their own antibiotics. Without such resistance mechanisms, the actinomycetia would be susceptible to self-toxicity caused by the synthesized antibiotics [120]. Antibiotic-resistance-guided screening is another novel method for discovering antibacterial compounds. Previously, this method was used to boost the production of aminoglycosides and lincosamides [121,122]. One research team used the resistance-based screening approach to find ansamycins using rifamycin resistance. Their study discovered high hit rates from glycopeptide antibacterials screening and identified several ansamycin producers [123]. RAST based evaluation of the whole-genome-sequenced actinomycetia strains revealed that the strains PBR1, PBR36, and DBR11 carried genes encoding fluroquinolone resistance. Additionally, the beta-lactam-resistant genes were present in the conserved regions of the genome of the *Streptomyces* sp. PBR1 strain.

## 5. Conclusions

In this work, both the genome mining and GC-MS analysis of all three actinomycetia strains, namely *Streptomyces* sp. DBR11, *Streptomyces* sp. PBR1, and *Streptomyces* sp. PBR36, revealed a diverse group of biologically active chemical classes with therapeutic potential, respectively. GC-MS analysis revealed that all three potent strains displayed diverse groups of chemical compounds, like heterocyclic compounds, phenyl, steroid, glycosides, amides, and ketones. These are often referred to as therapeutically important chemical classes, responsible for significant antimicrobial, antitumor, antimalaria, antituberculosis, anticancer, and anti-inflammatory activities. Similarly, the WGS data of the three potent strains also revealed that they contain a diverse group of BGCs associated with the biosynthesis of biologically important secondary metabolites with high therapeutic applications. In addition, the low MIC values and the SEM analysis of the pathogen’s cell surface morphology after treatment with the extract from three strains indicated that they may be promising antimicrobial candidates against drug-resistant human pathogens. The results of this study further support the significance of these forest-derived actinomycetia strains for future drug discovery. Overall, the findings emphasize the need for a comprehensive examination of the secondary metabolites produced by *Streptomyces*, as this could potentially lead to the discovery of novel pharmaceutical compounds.

## Figures and Tables

**Table 1:** MIC (µg/mL) of the ethyl acetate extract of three potent actinomycetia strains by broth dilution method.

Pathogens			
MIC (µg/mL)	MRSA (Gram-Positive Bacteria)	*Escherichia coli* GNR19 (Gram-Negative Bacteria)	*Candida albicans* (Yeast)
MIC of EtAc-DBR11	≤3.125	≤12.50	≤0.97
MIC of EtAc-PBR1	≤0.195	≤3.125	≤0.781
MIC of EtAc-PBR36	≤0.781	≤.781	≤0.097
MIC of Rifampicin	≤25	NA	NA
MIC of Amphotericin	NA	NA	≤0.97
MIC of Levofloxacin	NA	≤8	NA

Two-fold serial dilution of the extracts (working solution; 50 to 0.097 μg/mL) were prepared for MIC tests: medium used—nutrient agar for bacteria and potato dextrose broth for *Candida albicans*. EtAc: ethyl acetate extract; NA: not applicable; GNR19: MDR pathogen (clinical isolate).

**Table 2:** Chemical compounds detected in the three *Streptomyces* sp. strains by GC-MS analysis: (**A**) Chemical compounds detected in *Streptomyces* sp. PBR1. (**B**) Chemical compounds detected in *Streptomyces* sp. PBR36. (**C**) Chemical compounds detected in *Streptomyces* sp. DBR11.

Compound Name	RT	MW g/mol	Area (%)	Nature of Compound	Activity	References
(**A**)						
5-Amino-6-methoxy-8-nitroquinoline	8.688	204.18	4.44	Aromatic compound	No activity reported	-
Isonipecotic acid, N-[3-fluoro-5-trifluoromethylbenzoyl]-, eicosyl ester	11.526	129.16	3.23	Heterocyclic compound	Potential anticonvulsants	[42]
Acetic acid, hydrazino-, ethyl ester	20.25	131.13	2.43	Ethyl ester	No activity reported	-
4-Bromo-2-trifluoromethylphenyl isothiocyanate	24.905	282.08	2.42	Phenyl	Inhibitor of *Zika* virus infection, antiviral	[43]
Haloperidol TMS derivative	26.035	448	3.31	Organofluoride compound	Vasodilator activity, novel potent calcium channel blockers	[44]
Spiro [5.5] undecane, 1-methylene	28.725	164.29	3.77	Alkene	Anticancer	[45]
9-Acridanone, 1-ethoxy-3-methoxy-10- methyl-	32.732	283.32	2.37	Ketone	Anticancer, antimicrobial, antiviral, antimalarial, and anti-inflammatory activities	[46]
1-Anthracenyl-2-pyridyl ketone	36.602	283.30	3.94	Ketone	No activity reported	-
Fluoxymesterone	53.633	336.44	1.7	Steroid	Anticancer, antianemia	[47,48,49]
(**B**)						
Resibufogenin	28.727	384.23	1.15	Glycosides	Anticancer, anti-inflammation	[50]
o-Veratramide	33.365	181.19	1.21	Amide	Antimicrobial	[51]
5-Dicyanomethylene-9-diethylamino-6-fluorobenzo[a] Phenoxazine	38.311		4.17	heterocyclic compound	Antitumor activity, antimalaria, anti-*M. tuberculosis* activities	[52,53,54]
Lanostan-3. β. -ol, 11. beta.,19-epoxy	45.275	612.66	1.48	Alcohol	Anti-inflammatory	[55]
(**C**)						
2-[4-Bromo-phenyl]-indolizine	11	272.14	4.58	Phenyl	No activity reported	-
2-Bromo-N-[4-bromo-2-[2-bromo-benzoyl]-phenyl]-acetamide	13.242	387.10	1.92	Phenyl	No activity reported	-
D: A-Friedooleanan-3-ol, [3.alpha.]	13.479	428.73	3.64	Alcohol	No activity reported	-
Cyclopentanecarboxylic acid,1-[4-ethoxy-4-oxo-2-butenyl]-2-oxo-, ethyl ester	17.927	114.14	2.86	Aliphatic cycloalkyl carboxylic acid	No activity reported	-
L-Cysteine, N-acetyl-, methyl ester, acetate	21.396	163.20	4.9	Amino acid	Mucolytic agent and antioxidant	[56]
Desflurane	31.904	168.03	1.33	Fluorinated methyl ethyl ether	General anesthesia	[57]

RT—retention time; MW—molecular weight.

**Table 3:** (**A**) Overview of the predicted secondary metabolites from BGCs of *Streptomyces* sp. PBR1 by antiSMASH: (**B**) Overview of the predicted secondary metabolites from BGCs of *Streptomyces* sp. PBR36 by antiSMASH. (**C**) Overview of the predicted secondary metabolites from BGCs of *Streptomyces* sp. DBR11 by antiSMASH.

Category	Location	BGC Product	Type of Metabolite	Similarity %	MIBiG Accession
(**A**)					
Antibiotics	2-1,16,183	Lankamycin	Polyketide	60	BGC0000085
	49,267-1,31,743	Streptovaricin	Polyketide	51	BGC0001785
	908-23023	Lankacidin C	NRP + Polyketide	13	BGC0001100
	5447-39,902	Stenothricin	NRP: Cyclic depsipeptide	13	BGC0000431
	996-11,796	Chaxamycin A/chaxamycin B/chaxamycin C/chaxamycin D	Polyketide	6	BGC0001287
	27,195-61,444	Glycinocin A	NRP	6	BGC0000379
	40,809-55,207	Albaflavenone	Terpene	100	BGC0000660
	1-47,714	Murayaquinone	Polyketide	12	BGC0001675
	1-31,415	Granaticin	Polyketide: Type II	18	BGC0000227
	1-10,268	Ficellomycin	NRP	3	BGC0001593
	1-22,251	Aurantimycin A	NRP + Polyketide	27	BGC0001519
	1-7132	Merochlorin A/merochlorin B/deschloro-merochlorin A/deschloro-merochlorin B/isochloro-merochlorin B/dichloro-merochlorin B/merochlorin D/merochlorin C	Terpene + Polyketide: Type III	4	BGC0001083
	1-10,563	Lankacidin C	NRP + Polyketide	33	BGC0001100
	66,849-107,889	Violapyrone B	Polyketide	28	BGC0001905
	32,384-44,162	Desferrioxamin B/desferrioxamine E	Other	83	BGC0000940
Bioactive compounds	31,305-96,452	4-Z-annimycin	Polyketide	66	BGC0001298
	2683-82,260	Vazabitide A	NRP	34	BGC0001818
	11,961-45,511	4-hexadecanoyl-3-hydroxy-2-(hydroxymethyl)-2H-furan-5-one	Polyketide	54	BGC0000140
	2675-13,079	Ectoine	Other	100	BGC0000853
	10,103-31,335	Foxicins A-D	NRP + Polyketide	9	BGC0001598
	1-24,271	Polyoxypeptin	NRP + Polyketide	13	BGC0001036
	7948-22,853	Ashimides	NRP	12	BGC0001961
Others	93,612-1,16,183	Keywimysin	RiPP	100	BGC0001634
	1,11,462-1,26,572	Carotenoid	Terpene	45	BGC0000633
	45,047-66,372	2-methylisoborneol	Terpene	100	BGC0000658
	1-39,248	Flaviolin	Other	75	BGC0000902
	12,804-39,541	hopene	Terpene	84	BGC0000663
	12,735-35,872		lanthipeptide-class-II		
	8103-29,137		furan		
	1-6121		RiPP-like		
	1-11,690		RiPP-like		
	1-26,733		NAPAA		
	1-4266		RiPP-like		
(**B**)					
Antibiotics	19,357-40,370	Albaflavenone	Terpene	100	BGC0000660
	1-29,364	Stenothricin	NRP: Cyclic depsipeptide	13	BGC0000431
	60,663-88,764	Actinomycin D	NRP	64	BGC0000296
	55,417-76,448	Abyssomicin C	Polyketide: Modular type I	10	BGC0000001
	1-38,686	Ulleungmycin	NRP	16	BGC0001814
	1-62,481	Streptovaricin	Polyketide	21	BGC0001785
	1-57,823	Streptovaricin	Polyketide	39	BGC0001785
	1-47,111	Griseoviridin	NRP: Cyclic depsipeptide + Polyketide: Trans-AT type I	5	BGC0000459
	1-32,136	Tylactone	Polyketide	27	BGC0001812
	1-20,847	Aurantimycin A	NRP + Polyketide	24	BGC0001519
	159-10,665	Istamycin	Saccharide	4	BGC0000700
Bioactive compounds	58,700-79,758	Ebelactone	Polyketide	5	BGC0001580
	54,558-75,685	5-isoprenylindole-3-carboxylate Î^2^-D-glycosyl ester	Other	23	BGC0001483
	29,077-40,846	Desferrioxamin B/desferrioxamine E	Other	83	BGC0000940
	1-61,857	Vazabitide A	NRP	34	BGC0001818
	7767-17,982	Informatipeptin	RiPP: Lanthipeptide	42	BGC0000518
	986-11,384	Ectoine	Other	100	BGC0000853
	1-25,561	Polyoxypeptin	NRP + Polyketide	21	BGC0001036
	1-24,099	Polyoxypeptin	NRP + Polyketide	10	BGC0001036
Others	71,394-1,43,942	Spore pigment	Polyketide	66	BGC0000271
	19,226-41,835	SapB	RiPP: Lanthipeptide	100	BGC0000551
	1,08,091-1,34,835	Hopene	Terpene	100	BGC0000663
	13,597-64,516	Coelichelin	NRP	90	BGC0000325
	28,355-51,079	Germicidin	Other	100	BGC0001454
	7767-17,982	Geosmin	RiPP: Lanthipeptide	42	BGC0001181
	1-17,514	Isorenieratene	Terpene	100	BGC0000664
	89,467-1,00,801		RiPP-like		
	20,798-32,714		Siderophore		
	26,852-33,898		Siderophore		
(**C**)					
Antibiotics	1-33,814	Carbapenem MM4550	NRPS	6	BGC0000842
	20,764-41,777	Albaflavenone	Terpene	100	BGC0000660
	1-49,169	Borrelidin	Polyketide: Modular type I	4	BGC0000031
	17,271-45,046	Hormaomycin/Hormaomycin A1/Hormaomycin A2/Hormaomycin A3/Hormaomycin A4/Hormaomycin A5/Hormaomycin A6	NRP: Cyclic depsipeptide	13	BGC0000374
	1-14,775	Niphimycins C-E	Polyketide	6	BGC0001700
	30,422-36,402	Enduracidin	NRP	4	BGC0000341
	1-24,897	Stenothricin	NRP: Cyclic depsipeptide	18	BGC0000431
	1-2310	Icosalide A	NRP: Lipopeptide	100	BGC0001833
Bioactive compounds	522-41,646	Herboxidiene	Polyketide	8	BGC0001065
	1-25,422	Undecylprodigiosin	NRP+ Polyketide	72	BGC0001063
	141-66,426	Ashimides	NRP	100	BGC0001961
	11,063-22,844	Desferrioxamin B/desferrioxamine E	Other	83	BGC0000940
	16,419-26,817	Ectoine	Other	100	BGC0000853
	7496-27,381	Chejuenolide A/Chejuenolide B	Polyketide	7	BGC0001543
	5274-22,349	5-isoprenylindole-3-carboxylate Î^2^-D-glycosyl ester	Other	28	BGC0001483
	941-8166	Informatipeptin	RiPP: Lanthipeptide	28	BGC0000518
	1-2005	Rhizomide A/Rhizomide B/Rhizomide C	NRP	100	BGC0001758
	1-1546	Rhizomide A/Rhizomide B/Rhizomide C	NRP	100	BGC0001758
Others	36,474-62,012	Isorenieratene	Terpene	100	BGC0001456
	10,186-62,421	Coelibactin	NRP	100	BGC0000324
	39,729-98,004	Germicidin	Other	100	BGC0001454
	18,828-41,614	SapB	RiPP: Lanthipeptide	100	BGC0000551
	11,789-38,527	Hopene	Terpene	100	BGC0000663
	2958-13,551	Melanin	Other	60	BGC0000909
	1-10,206	Spore pigment	Polyketide	58	BGC0000271
	37,835-58,260		Phenazine		
	1741-24,817		lanthipeptide-class-I		
	1-7577		RiPP-like		
	1-7342		butyrolactone		
	1-7156		NRPS		
	1-1627		NRPS		
	1-1627		NRPS		

## Data Availability

The data presented in this study are available in the main text and supplementary Table S5.

## Supplementary Materials

The following supporting information can be downloaded at https://www.mdpi.com/article/10.3390/metabo13080911/s1, Figure S1: GC-MS analysis of the ethyl acetate extracts of actinomycetia strains: Chemical structures of the identified compounds (I) *Streptomyces* sp. PBR1; (II) *Streptomyces* sp. PBR36; and (III) *Streptomyces* sp. DBR11; Table S1: In vitro sensitivity profile of MDR pathogens against standard antibiotics; Table S2: Morphological characteristics of 65 actinomycetia isolates with their respective media and place of isolation; Table S3: Presence of biosynthetic genes (PKS II and NRPS) and antimicrobial activity of the representative isolates of actinomycetia; Table S4: Antimicrobial activity of actinomycetia isolates by well diffusion method against MDR pathogens; Table S5: Molecular identification of antagonistic actinomycetia based on 16S rRNA gene sequences; Table S6: WGS assembly statistics, quality report, and taxonomic identification of *Streptomyces* sp. DBR11, *Streptomyces* sp. PBR1, and *Streptomyces* sp. PBR36.
