# Supplementary material for: Potentiality of Actinomycetia Prevalent in Selected Forest Ecosystems in Assam, India to Combat Multi-Drug-Resistant Microbial Pathogens
Source: Metabolites. 2023 Aug 3;13(8):911. doi: 10.3390/metabo13080911 (PMC10456813; doi:10.3390/metabo13080911)
Supplement: Supplementary file 1 [file metabolites-13-00911-s001.zip › supplementary figure.pdf]

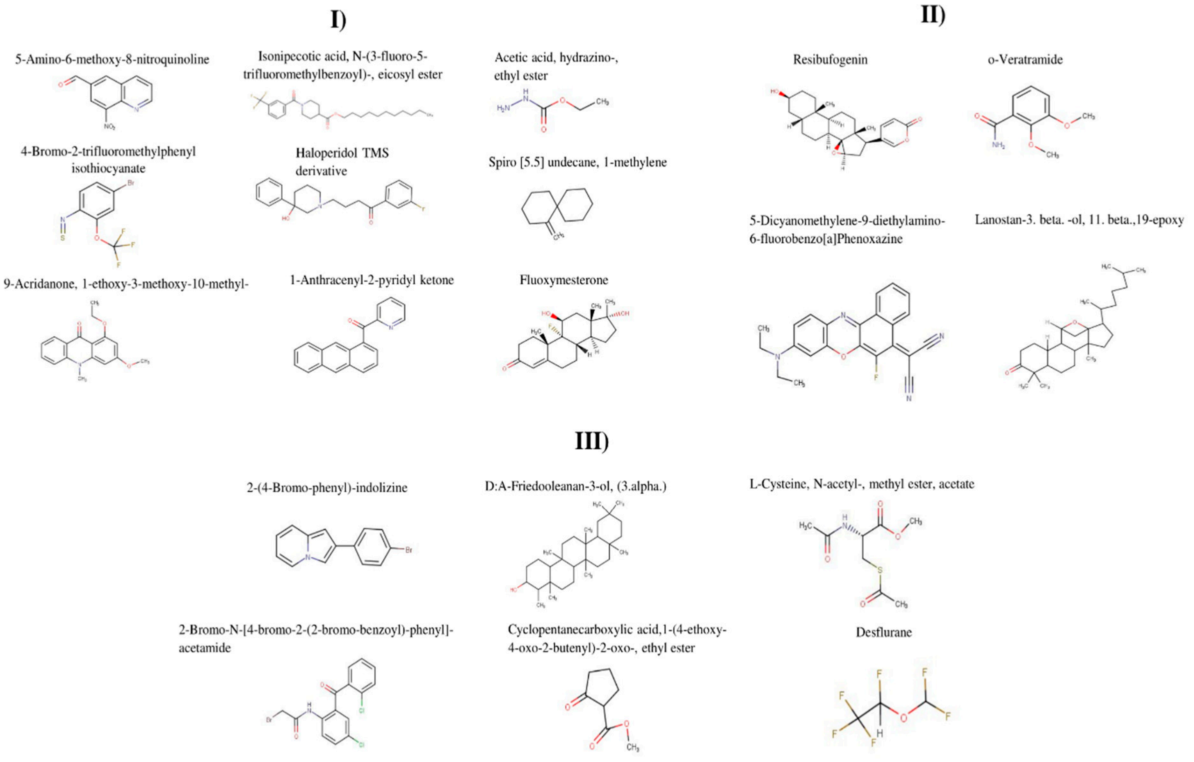

Figure S1. GC-MS analysis of the ethyl acetate extracts of actinomycetia strains. Chemical structures of the identified compounds (I) *Streptomyces* sp. PBR1; (II) *Streptomyces* sp. PBR36; (III) *Streptomyces* sp. DBR11.
