# Supplementary material for: Potentiality of Actinomycetia Prevalent in Selected Forest Ecosystems in Assam, India to Combat Multi-Drug-Resistant Microbial Pathogens
Source: Metabolites. 2023 Aug 3;13(8):911. doi: 10.3390/metabo13080911 (PMC10456813; doi:10.3390/metabo13080911)
Supplement: Supplementary file 1 [file metabolites-13-00911-s001.zip › Supplementary tables.pdf]

Table S1. ***In vitro*** **sensitivity profile of MDR pathogens against standard antibiotics.**

**Sample ID**	**Colony Color**	**Causative pathogen (Preliminary screening)**	**Ampicillin (AMP) 10 µg**	**Cefazolin (CZ) 30 µg**	**Nalidixic acid (NA) 30 µg**	**Norfloxacin (NX) 10 µg**	**Ciprofloxacin (CIP) 5 µg**	**Co-trimoxazole (COT) 25 µg**	**Levofloxacin (LE) 5 µg**	**Nitrofurantoin (NIT) 300 µg**
GNR1	Pink	*E. coli*	R	R	R	I	R	R	I	15
GNR2	Blue	*E. faecalis*	R	R	R	25	I	R	30	R
GNR3	Purple	*K. pneumoniae*	R	R	R	R	R	R	R	18
GNR4	Blue	*E. faecalis*	R	17	R	I	R	20	R	10
GNR5	Blue	*E. faecalis*	20	17	R	R	R	R	R	17
GNR6	White	*C. albicans*	20	26	R	17	25	22	15	R
GNR7	Blue	*E. faecalis*	R	R	R	I	R	R	R	I
GNR8	Pink	*E. coli*	R	R	R	I	R	R	I	R
GNR9	Pink	*E. coli*	R	R	R	R	R	R	R	R
GNR10	White	*C. albicans*	I	25	18	18	18	18	20	13
GNR11	Blue	*E. faecalis*	R	R	R	I	R	35	R	R
GNR12	Pink	*E. coli*	R	R	R	I	R	R	R	R
GNR13	Pink	*E. coli*	R	R	R	I	R	25	R	18
GNR14	Purple	*K. pneumonia*	14	23	R	R	R	R	R	28
GNR15	Pink	*E. coli*	R	R	R	22	R	R	R	R
GNR16	Pink	*E. coli*	R	R	R	21	R	R	I	R
GNR17	Blue	*E. faecalis*	R	21	19	23	I	22	29	R
GNR18	Yellow	*P. aeruginosa*	R	26	R	R	R	R	R	I
GNR19	Pink	*E. coli*	R	R	R	R	R	R	R	R

Antimicrobial susceptibility test as per CLSI criteria; 2020. Inhibition zone in mm. (**R**-Resistant, **I**- Intermediate)

Table S2. Morphological characteristics of 65 actinomycetia isolates with their respective media and place of isolation.

Sl. No	Isolate code	Isolation site	Isolation media	Color of aerial mycelium	Color of substrate mycelium	Diffusible pigment	Colony morphology [Form, margin, elevation]
1.	PBR1	PWS	AIA	Pale yellow	Light brown	NP	Irregular, undulate, raised
2.	PBR2	PWS	SA	Brown	Light brown	NP	Irregular, lobate, raised
3.	PBR3	PWS	AIA	Pale yellowish	Light yellow	NP	Irregular, undulate, flat
4.	PBR4	PWS	SA	Creamy brown	Light brown	NP	Irregular, undulate, umbonate
5.	PBR5	PWS	SA	Brown	Brown	NP	Circular, undulate, convex
6.	PBR6	PWS	SA	Creamy white	Creamy white	NP	Irregular, curled, pulvinate
7.	PBR7	PWS	SA	Light brown	Pale yellow	NP	Circular, undulate, flat
8.	PBR8	PWS	SA	White	Pale yellow	Yellowish	Circular, lobate, umbonate
9.	PBR9	PWS	SA	Creamy white	Light brown	NP	Irregular, undulate, flat
10.	PBR10	PWS	SA	Creamy white	Creamy white	NP	Circular, lobate, umbonate
11.	PBR11	PWS	AIA	White	Pale yellow	NP	Irregular, flat, undulate
12.	PBR12	PWS	AIA	Creamy white	Creamy white	NP	Irregular, undulate, raised
13.	PBR13	PWS	SA	Pinkish white	Pale yellow	Yellowish	Circular, undulate, umbonate
14.	PBR14	PWS	SA	Creamy white	white	NP	Irregular, lobate, umbonate
15.	PBR15	PWS	SA	Creamy white	Creamy white	NP	Irregular, curled, convex
16.	PBR16	PWS	SA	Pale yellow	Yellowish	Yellowish	Circular, undulate, umbonate
17.	PBR17	PWS	SA	Pink	Pink	Brownish	Circular, undulate, raised
18.	PBR18	PWS	SA	Light brown	Light brown	NP	Circular, undulate, umbonate
19.	PBR19	PWS	SA	Grey white	Whitish	NP	Circular, undulate, raised
20.	PBR20	PWS	AIA	Creamy brown	Light brown	NP	Circular, undulate, raised
21.	PBR21	PWS	AIA	Creamy white	Creamy white	NP	Circular, entire, convex
22.	PBR22	PWS	SA	White	Pale yellow	NP	Irregular, undulate, raised
23.	PBR23	PWS	SA	White	Creamy white	Brownish	Circular, undulate, raised
24.	PBR24	PWS	SA	Creamy white	Pale yellow	Pale yellow	Circular, undulate, flat
25.	PBR25	PWS	AIA	Light pink	Pink	NP	Circular, entire, convex
26.	PBR26	PWS	AIA	Pink	Pink	NP	Circular, undulate, raised
27.	PBR27	PWS	SA	Light brown	Brownish	NP	Circular, entire, umbonate
28.	PBR28	PWS	SA	Creamy brown	Creamy brown	NP	Irregular, undulate, umbonate
29.	PBR29	PWS	SA	Light brown	Brownish	NP	Circular, undulate, umbonate
30.	PBR30	PWS	SA	Creamy white	Creamy white	NP	Circular, entire, pulvinate
31.	PBR31	PWS	AIA	White	Pale yellow	NP	Circular, entire, convex
32.	PBR32	PWS	SA	Creamy white	Light brown	NP	Circular, entire, umbonate
33.	PBR33	PWS	SA	White	Dark brown	Brownish	Circular, entire, umbonate
34.	PBR34	PWS	SA	Dark brown	Dark brown	Brownish	Irregular, undulate, umbonate
35.	PBR35	PWS	AIA	Light yellow	yellow	Yellowish	Circular, undulate, crateriform
36.	PBR36	PWS	AIA	White	Light brown	Brownish	Circular, undulate, umbonate
37.	PBR37	PWS	SA	Yellow	Pale yellow	NP	Circular, undulate, umbonate
38.	PBR38	PWS	SA	Creamy white	Pale yellow	NP	Circular, undulate, umbonate
39.	PBR39	PWS	AIA	Black	Black	NP	Circular, undulate, raised
40.	DBR1	DBWS	AIA	Pale yellow	Light brown	NP	Irregular, undulate, raised
41.	DBR2	DBWS	AIA	Dark brown	Dark brown	Brownish	Circular, undulate, umbonate
42.	DBR3	DBWS	SA	Greyish white	Dark brown	NP	Circular, undulate, raised
43.	DBR4	DBWS	AIA	Creamy white	Creamy white	NP	Circular, undulate, umbonate
44.	DBR5	DBWS	SA	Creamy white	Creamy white	NP	Circular, undulate, umbonate
45.	DBR6	DBWS	SA	White Brownish	Brownish	NP	Irregular, undulate, umbonate
46.	DBR7	DBWS	AIA	White Brownish	Brownish	NP	Irregular, undulate, umbonate
47.	DBR8	DBWS	SA	Dark Brownish	Brownish	NP	Circular, undulate, umbonate

48.	DBR9	DBWS	SA	Pinkish white	Pinkish	NP	Circular, Rough, Convex,
49.	DBR10	DBWS	SA	Pinkish	Pinkish	NP	Irregular, granulated, raised
50.	DBR11	DBWS	AIA	Greyish pink	Pinkish	NP	Circular, undulate, raised
51.	DBR12	DBWS	AIA	Orange	Orange	NP	Powdery, circular, flat
52.	DBR13	DBWS	AIA	Whitish orange	Whitish orange	NP	Circular, undulate, raised
53.	DBR14	DBWS	SA	White	Creamy white	NP	Rough, Convex, irregular
54.	DBR15	DBWS	SA	White	Creamy white	NP	Circular, undulate, umbonate
55.	DBR16	DBWS	SA	Brown pink	Brown pink	NP	Circular, undulate, umbonate
56.	DBR17	DBWS	SA	Dark brown	Brownish	NP	Circular, convex, umbonate
57.	DBR18	DBWS	SA	Dark brown	Dark brown	Brownish	Circular, undulate, umbonate
58.	DBR19	DBWS	SA	White	Brownish	Brownish	Circular, lobate, umbonate
59.	DBR20	DBWS	SA	Light brown	Yellowish	NP	Circular, entire, convex,
60.	DBR21	DBWS	SA	Whitish pink	Dark pink	NP	Circular, lobate, umbonate
61.	DBR22	DBWS	SA	Light Orange	Light Orange	NP	Irregular, flat, lobate
62.	DBR23	DBWS	AIA	White	Pink white	NP	Circular, undulate, umbonate
63.	DBR24	DBWS	AIA	Pinkish	Pinkish	NP	Circular, flat, entire
64.	DBR25	DBWS	SA	Orange	Orange	NP	Circular, lobate, flat
65.	DBR X	DBWS	AIA	Orange	Orange	NP	Circular, undulate, flat,

PWS: Pobitora Wildlife Sanctuary; **DBWS**: Deepor Beel Wildlife Sanctuary; **NP**: Not pigmented; **SA**: Streptomyces agar media; **AIA**: Actinomycetes Isolation Agar media.

Table S3. Presence of biosynthetic genes (PKSII and NRPS) and antimicrobial activity of the representative isolates of actinomycetia

Strain name	Inhibition zone (mm)				Biosynthetic gene	
	MRSA	*Klebsiella pneumoniae*	*Pseudomonas aeruginosa*	*Candida albicans*	*NRPS*	*PKS-II*
PBR36	$24.33^{ab} \pm 0.33$	$27.33^{ab} \pm 0.33$	$24.67^{ab} \pm 0.33$	$26.67^{afgn} \pm 0.33$	+	+
DBR10	$24.00^{ab} \pm 0.58$	$23.00^{ab} \pm 0.58$	$34.33^{a} \pm 0.33$	$14.67^{abg} \pm 0.33$	ND	+
DBR11	$19.67^{ab} \pm 0.33$	$21.67^{ab} \pm 0.33$	$21.00^{ac} \pm 0.58$	$20.33^{afn} \pm 0.33$	+	+
DBR25	$21.33^{abc} \pm 0.88$	$24.67^{ab} \pm 0.33$	$17.00^{abd} \pm 0.58$	$26.33^{afn} \pm 0.33$	ND	ND
PBR1	$30.67^{a} \pm 0.33$	$35.00^{a} \pm 0.58$	$29.00^{ab} \pm 0.58$	$22.33^{afgn} \pm 0.33$	+	+
PBR11	$29.00^{a} \pm 1.00$	$30.33^{a} \pm 0.33$	$21.00^{ab} \pm 1.00$	$47.00^{afgn} \pm 1.00$	ND	+
PBR30	$18.33^{ab} \pm 0.33$	$20.33^{ab} \pm 0.33$	$18.00^{abd} \pm 0.58$	$24.33^{c} \pm 0.33$	+	+
PBR35	$19.00^{ab} \pm 0.58$	$24.33^{ab} \pm 0.33$	$15.00^{abd} \pm 0.58$	$18.33^{adg} \pm 0.33$	+	+
PBR4	$28.67^{ab} \pm 0.88$	$13.67^{b} \pm 0.33$	$20.00^{ab} \pm 0.58$	$14.33^{aeg} \pm 0.33$	ND	+
DBR3	$14.33^{b} \pm 0.33$	NA	$10.67^{b} \pm 0.33$	$15.00^{afghn} \pm 0.58$	ND	+
DBR17	NA	$21.00^{ab} \pm 0.58$	$19.33^{bc} \pm 0.33$	19.67afgn ± 0.33	ND	ND
PBR21	$24.00^{ab} \pm 0.58$	NA	$22.33^{ab} \pm 0.33$	$34.33^{cfl} \pm 0.33$	ND	+
DBRX	$23.00^{ab} \pm 0.58$	NA	$20.00^{ab} \pm 0.58$	$9.67^{ghmo} \pm 0.33$	+	+
DBR1	$10.00^{abc} \pm 0.58$	$14.33^{bd} \pm 0.33$	$24.33^{ab} \pm 0.33$	NA	ND	+
DBR33	$21.33^{abc} \pm 0.88$	NA	NA	NA	ND	+
PBR19	$22.33^{ab} \pm 0.33$	NA	NA	NA	ND	+
DBR21	NA	NA	$22.67^{ab} \pm 0.33$	NA	ND	+
PBR16	NA	NA	NA	$19.67^{almo} \pm 0.33$	ND	+
DBR5	NA	NA	NA	$15.33^{bdeno} \pm 0.33$	ND	+

Zone of inhibition by well diffusion method. Zone of inhibition values are given as mean ± standard error of mean (n = 3). Values in the same row and sub table not sharing the same superscript are significantly different at $p < .001$; **NA**- No activity; **ND**- Not detected; **+,** Detected. **NRPS**; Nonribosmal peptide synthetases, **PKS-II**; Type II polyketide synthases

Table S4. Antimicrobial activity of actinomycetia isolates by well diffusion method against MDR pathogens.

**Strain name**	**Inhibition zone (mm)**		
	**GNR7 (*Enterococcus faecalis*)**	**GNR18 (*Pseudomonas aeruginosa*)**	**GNR19 (*Escherichia coli*)**
PBR36	$29.67^{a} \pm 0.33$	$24.67^{a} \pm 0.33$	$24.67^{ac} \pm 0.33$
DBR10	$24.33^{ac} \pm 0.33$	$16.33^{ah} \pm 0.33$	$24.33^{ac} \pm 0.33$
DBR11	$30.00^{ac} \pm 0.58$	$20.33^{ah} \pm 0.33$	$22.33^{ac} \pm 0.33$
DBR25	$21.00^{ac} \pm 0.58$	$22.00^{ah} \pm 0.58$	$21.00^{ac} \pm 0.58$
PBR1	$22.00^{ac} \pm 0.58$	$27.33^{a} \pm 0.33$	$23.67^{ac} \pm 0.33$
PBR11	NA	$25.33^{a} \pm 0.33$	$29.33^{a} \pm 0.33$
PBR30	$20.33^{ac} \pm 0.33$	$16.00^{ab} \pm 0.58$	$20.67^{ac} \pm 0.33$
PBR35	$14.67^{c} \pm 0.33$	$20.33^{ah} \pm 0.33$	$20.33^{ac} \pm 0.33$
PBR4	NA	$14.33^{ah} \pm 0.33$	$19.67^{ac} \pm 0.33$
DBR3	NA	NA	$22.67^{ac} \pm 0.33$
DBR17	$15.00^{abc} \pm 0.58$	NA	$23.67^{ac} \pm 0.33$
PBR21	NA	$20.67^{ah} \pm 0.33$	$24.67^{ac} \pm 0.33$
DBRX	NA	NA	$20.33^{ac} \pm 0.33$
DBR1	NA	NA	$20.33^{ac} \pm 0.33$
DBR33	$21.00^{ac} \pm 0.58$	NA	NA
PBR19	$20.33^{ac} \pm 0.33$	NA	NA
DBR21	$21.00^{ac} \pm 0.58$	$8.33^{bhi} \pm 0.33$	$22.67^{ac} \pm 0.33$
PBR16	NA	$14.67^{ai} \pm 0.33$	$14.33^{c} \pm 0.33$
DBR5	$11.00^{abc} \pm 0.58$	NA	NA

(Zone of inhibition by well diffusion method. Zone of inhibition values are given as mean ± standard error of mean (n = 3). Values in the same row and sub table not sharing the same superscript are significantly different at $p < .001$, **NA-** No activity)

Table S5. Molecular dentification of antagonistic actinomycetia based on 16S rRNA gene sequences

Isolate Name	NCBI-GenBank accession number	Length (bp)	Top-hit taxon name with accession number	Similarity %	Species identification
PBR36	MH922854	1,334	*Streptomyces parvulus* AB184326	100	*Streptomyces* sp.
DBR10	MH922850	1,361	*Streptomyces ardesiacus* DQ026631	98.01	*Streptomyces* sp.
DBR11	MH922859	1,339	*Streptomyces ardesiacus* DQ026631	99.63	*Streptomyces* sp.
DBR25	MH922861	1,339	*Nonomuraea wenchangensis* FJ261959	98.88	*Nonomuraea* sp.
PBR1	MH922852	1,312	*Streptomyces kunmingensis* AB184597	99.08	*Streptomyces* sp.
PBR30	MH922863	1,355	*Streptomyces philanthi* DQ375802	98.93	*Streptomyces* sp.
PBR35	MN069557	1,389	*Streptomyces parvulus* AB184326	99.93	*Streptomyces* sp.
PBR4	MH922849	1,341	*Streptomyces kebangsaanensis* HM449824	98.95	*Streptomyces* sp.
DBR3	MH922864	1,345	*Streptomyces kebangsaanensis* HM449824	98.81	*Streptomyces* sp.
PBR21	MH922856	1,210	*Streptomyces racemochromogenes* DQ026656	99.17	*Streptomyces* sp.
DBR17	MH922855	1,346	*Actinomadura nitritigenes* AY035999	99.26	*Actinomadura* sp.
DBR1	MH922858	1,310	*Streptomyces parvulus* AB184326	99.85	*Streptomyces* sp.
DBR33	MH922857	1,330	*Streptomyces deserti* HE577172	98.79	*Streptomyces* sp.
PBR19	MH922862	1,348	*Streptomyces atrovirens* DQ026672	93.96	*Streptomyces* sp.
DBRX	MH922853	1,303	*Nocardia nova* BDBN01000167	99.92	*Nocardia* sp.
PBR11	MH718314	1,370	*Streptomyces atrovirens* DQ026672	92.91	*Streptomyces* sp.
DBR5	MH922860	1,343	*Streptomyces parvulus* AB184326	99.25	*Streptomyces* sp.
DBR21	MK981152	1,364	*Streptomyces rubrogriseus* AJ781373	99.63	*Streptomyces* sp.
DBR16	MH922851	1,371	*Streptomyces ardesiacus* DQ026631	99.64	*Streptomyces sp.*

Table S6. WGS assembly statistics, quality report and taxonomic identification of *Streptomyces* sp. DBR11, *Streptomyces* sp. PBR1 and *Streptomyces* sp. PBR36

Features		Strain name		
		***Streptomyces sp.* DBR11**	***Streptomyces* sp. PBR1**	***Streptomyces* sp. PBR36**
**ANI Taxonomic identification**		*Streptomyces coelicoflavus*	*Streptomyces longispororuber*	*Streptomyces parvulus*
**BioSample**		SAMN28084415	SAMN28084416	SAMN28084415
**BioProject number**		PRJNA834923	PRJNA834923	PRJNA834923
**SRA**		SRX15248015	SRX15248016	SRX15248017
**Scaffolds**		378	448	359
**Total length**		8076063	9557226	7334189
**Gaps**		0	0	0
**N50 [Kb]**		39	38	34
**GC [%]**		72.09	71.75	72.82
**No. of protein-coding genes**		7218	9018	6250
**CRISPR**		1	2	-
**rRNA**		3	3	3
**tRNA**		75	87	76
**tmRNA**		1	1	1
**BGC**		31	33	29
**BUSCO Analysis**	**Complete [%]**	98.4	97.7	98.7
	**Fragmented [%]**	0.8	0.4	0.6
	**Missing [%]**	0.8	1.9	0.7
